# The SAFE Model: State Authenticity as a Function of Three Types of Fit

**DOI:** 10.1177/01461672231223597

**Published:** 2024-01-28

**Authors:** Audrey Aday, Yingchi Guo, Smriti Mehta, Serena Chen, William Hall, Friedrich M. Götz, Constantine Sedikides, Toni Schmader

**Affiliations:** 1 The University of British Columbia, Vancouver, Canada; 2 University of California, Berkeley, USA; 3 Brock University, St. Catharines, Ontario, Canada; 4 University of Southampton, UK

**Keywords:** authenticity, fit, social belonging, motivation, experience sampling methodology

## Abstract

The SAFE model asserts that state authenticity stems from three types of fit to the environment. Across two studies of university students, we validated instruments measuring self-concept, goal, and social fit as unique predictors of state authenticity. In Study 1 (*N =* 969), relationships between fit and state authenticity were robust to controlling for conceptually similar and distinct variables. Using experience sampling methodology, Study 2 (*N* = 269) provided evidence that fit and authenticity co-vary at the state (i.e., within-person) level, controlling for between-person effects. Momentary variation in each fit type predicted greater state authenticity, willingness to return to the situation, and state attachment to one’s university. Each fit type was also predicted by distinct contextual features (e.g., location, activity, company). Supporting a theorized link to cognitive fluency, situations eliciting self-concept fit elicited higher working memory capacity and lower emotional burnout. We discuss the implications of fit in educational contexts.

People are motivated to feel authentic and true to themselves (Maslow, 1954; Rogers, 1961). Although authenticity was originally considered a disposition (Kernis & Goldman, 2005; Wood et al., 2008), it varies both between and within-person (Chen, 2019; Fleeson & Wilt, 2010; Landa & English, 2021; Lenton et al., 2016; Sedikides et al., 2019). State authenticity encompasses “the sense of feeling that one is currently in alignment with one’s true or genuine self” (Sedikides et al., 2017, p. 521). Given the benefits of feeling authentic for well-being (Hicks et al., 2019; Kifer et al., 2013; Lutz et al., 2023), a moral sense of self (Newman et al., 2014), and a sense of power (Gan et al., 2018; Kraus et al., 2011), people are understandably attracted to contexts where they feel authentic. Yet, predictors of state authenticity are not well established.

We sought to elucidate the role of person-environment fit in predicting state authenticity, which in turn guides one’s approach and avoidance of situations. We provide the first empirical test of key assumptions from the SAFE model, which asserts that state authenticity reflects three types of fit to the environment: self-concept, goal, and social fit (Schmader & Sedikides, 2018). Two studies using survey and experience sampling methods validate instruments to guide future investigations on when, why, and how people self-select into contexts that cue authenticity.

## The SAFE Model: Three Types of Fit to the Environment

The SAFE model (Schmader & Sedikides, 2018) conceptualizes state authenticity as a subjective state that is situated within, and activated by, the environment. State authenticity is not only experienced as a short-lived emotion but can be repeatedly experienced in a given context (e.g., feeling most like oneself when at home). People seek out situations that afford authenticity and avoid those that do not. Importantly, because contexts contain identity-relevant cues, the same setting can allow some individuals or groups to thrive while alienating others. According to the SAFE model, state authenticity arises from three distinct but interrelated types of fit between the self and environment: self-concept fit, goal fit, and social fit. The focus of the SAFE model is on the subjective experience of fit, not objective measures of correspondence between personality and situational characteristics (Fulmer et al., 2010; Götz et al., 2018).

### Self-Concept Fit

Self-concept fit is theorized to be the cognitive component of state authenticity. According to the SAFE model, self-concept fit occurs when the environment activates a familiar sense of self (Markus & Wurf, 1987). For example, when entering a sports arena, one’s self-concept as “sports fan” might be activated. Given that repeated activation of cognitions facilitates fluent processing, and cognitions processed fluently are judged to be true (Reber & Unkelbach, 2010), self-concept fit and corresponding feelings of cognitive fluency manifest as a sense of a “true self.” In contrast, a mismatch between the self and the environment will be cognitively disfluent, placing demands on executive attention and working memory capacity (Engle, 2002), and eliciting inauthenticity. Self-concept fit can be passively elicited from environmental cues and does not require active goal pursuit or the presence of others.

### Goal Fit

Goal fit is theorized to be the motivational component of state authenticity, occurring when environments facilitate motivational fluency toward self-relevant goals. People feel motivational fluency when task demands match their own orientation toward the task (Higgins, 2005). For example, students who prefer to learn through quiet reflection feel a greater goal fit when constructing a persuasive argument in writing versus group discussion (Kim, 2002). When individuals’ own goal orientation matches environmental affordances, their actions feel autonomous and authentic (Heppner et al., 2008). Thus, environments that cue goal fit and a corresponding sense of motivational fluency offer another route to state authenticity.

### Social Fit

Social fit is the social component of state authenticity and occurs when other people in the environment accept and validate one’s true self, facilitating interpersonal fluency. For example, an artist might feel social fit when their creativity is recognized and encouraged. The need to belong is a fundamental human need (Baumeister & Leary, 1995), and people automatically detect cues of acceptance or rejection (Pickett et al., 2004). People feel authentic when they are able to enact their true self in social interactions (Swann, 2012) and feel inauthentic when they try to gain social acceptance by conforming to others’ standards (Goffman, 1959; Lenton, Bruder, et al., 2013).

## The Benefits of Distinguishing Among Three Types of Fit

Because these three distinct predictors of state authenticity have not previously been empirically tested, validating an instrument to disentangle different routes to state authenticity could have several benefits. This does not mean that a given environment will only cue one type of fit, but rather that different types of fit can be theoretically and empirically distinguished. For example, one situation could involve being with similar others who both activate a default sense of self (high self-concept fit) and socially validate that self-view (high social fit), but another situation could signal a domain that activates a default sense of self (high self-concept fit) even though the people there do not validate that self-view (low social fit). Distinguishing among the cognitive, motivational, and social facets of fit can also help pinpoint environment-specific predictors of state authenticity. Self-concept fit might uniquely predict state authenticity in familiar environments, goal fit might uniquely predict authenticity when productivity is valued, and social fit might uniquely predict authenticity during social interactions with close others. We sought to validate distinct measures of fit that explain unique variance in state authenticity both across people in the same general environment (e.g., students’ experience at their university) and across different situations for the same person (e.g., daily experiences on campus).

Second, the three types of fit are extensions of related constructs. For example, belonging measures often capture a general feeling of *fit* (Mendoza-Denton et al., 2002), but have been used to assess interpersonal fit (Walton & Cohen, 2007), goal-relevant fit (Belanger et al., 2020), and ambient inclusion (Cheryan et al., 2009). A tripartite conception of fit provides greater theoretical specificity for these experiences and a validated measure that can distinguish among them. Relatedly, self-determination theory (SDT; Deci & Ryan, 1985) parses people’s needs for autonomy, relatedness, and competence that, when satisfied, promote authenticity (Heppner et al., 2008). Whereas SDT focuses on individual needs, the SAFE model emphasizes the dynamic fit between the environment and identity.

Third, the SAFE model hypothesizes that each type of fit predicts engagement with environments via state authenticity (Schmader & Sedikides, 2018). People gravitate toward environments that activate their default self-concept (self-concept fit; Cheryan et al., 2009; Matz & Harari, 2021), afford personally valued goals (goal fit; Diekman et al., 2017), and socially validate their self-views (social fit; Dasgupta, 2011; Leary & Kelly, 2009). People leave environments that fail to afford experiences of fit. For example, in educational and work settings, a lack of fit between one’s own values and those of the institution can predict lower performance and motivation (Cable & DeRue, 2002; Phillips et al., 2020; Stephens et al., 2012) with implications for a student’s intention to drop out of school (Suhlmann et al., 2018).

Our approach extends prior work not only by estimating the relative contribution of each type of fit to university students’ level of authenticity but also by identifying predictors and outcomes of students’ feelings of fit to their academic environment. In addition, the constructs and instruments we validate are intended to generalize beyond academic settings, with eventual applications to experiences of misfit felt by marginalized groups.

## Overview

We report two studies assessing students’ state authenticity and academic experiences as predicted by three distinct types of fit. In Study 1, we validated a new self-report instrument (i.e., the SAFE scale) that assesses each type of fit, and tested whether self-concept, goal, and social fit explained unique variation in undergraduates’ state authenticity and dropout intentions. In Study 2, we used an experience sampling paradigm to test whether: (a) students feel more authentic in campus situations that cue each type of fit, (b) different situational features uniquely predict each type of fit, and (c) within-person variation in fit predicts momentary outcomes including situation selection and state attachment to the university as well as working memory capacity and burnout (potential indicators of cognitive fluency). All studies were approved by the Institutional Review Board (IRB) of the University of British Columbia (Study 1 was also approved by the University of California Berkeley IRB).

## Study 1

### Pilot Study

We first conducted an online pilot study to develop SAFE scale items assessing self-concept, goal, and social fit experienced by employees in their organizations. Two of the authors generated an initial pool of 26 items derived from descriptions of each type of fit in Schmader and Sedikides (2018; see Supplementary Figures S1 and S2, Table S2), and 259 working adults on MTurk rated these items. An exploratory principal axis factor analysis with oblique, promax rotation (Costello & Osborne, 2005) suggested three discrete factors that aligned with the theoretical foundations of the SAFE model. Based on this analysis, we selected the five highest-loading items from each factor (all factor loadings > .40, all with cross-loadings below .30; Costello & Osborne, 2005), and created reliable composites for each type of fit (see Table 1).^1^

**Table 1.** Items on the State Authenticity as Fit to Environment (SAFE) Scale Assessing Self-Concept, Goal, and Social Fit in an Organizational (Pilot) and University Context (Study 1).

Fit type	Item wording
Self-concept fit Pilot: α = .94 Study 1: α = 94	Even when I’m alone and doing nothing, simply being at [university name/ company name] makes me feel like myself.
	Just being at [university name/ company name] suits the way I see myself.
	[University name/ company name] feels right for who I am.*
	Being at [university name/ company name] brings out who I am.*
	I feel ‘at home’ when I’m at [university name/ company name].
Goal fit Pilot: α = .91 Study 1: α = 86	[University name/ company name] is a place where I feel intrinsically motivated by my own goals.
	Standards of success at [university name/ company name] match what I think it means to be successful.
	I feel that [university name/ company name] is a place that allows me to realize my own goals.
	My behavior at [university name/ company name] is motivated by things I value.
	Classes at [university name]/ Tasks at [company name] are designed in a way that fits how I like to [learn/ work].
Social fit Pilot: α = .91 Study 1: α = 89	When I’m around [other students/ my coworkers] on campus, I feel like I can act natural.*
	I don’t feel like I need to be a different person around others at [university name/ company name].
	Other students at (university name/ Other coworkers at [company name) do NOT judge me for being myself.*
	I never have to hide my true behavior when I’m with others at [university name/ company name].*
	I feel that people at [university name/ company name] understand exactly who I am.

*Note.* In Study 1, we reworded five items (*) from the pilot study to exclude mention of “true self” intending to reduce conceptual overlap with the authenticity measure. Items were rated from 1 (*strongly disagree*) to 7 (*strongly agree*).

In Study 1, we modified and validated the SAFE scale to assess university students’ self-concept, goal, and social fit, and tested the hypothesis that all three types of fit would explain unique variability in dropout intentions, mediated by state authenticity. We established construct validity by testing the convergent validity of the SAFE scale with an undifferentiated measure of belonging. We also tested the discriminant validity of each fit from other distinct constructs (e.g., SDT constructs— Ryan & Deci, 2000; a sense of self—Flury & Ickes, 2007; goal motivation—Sedikides et al., 2019; and social belonging Yeager et al., 2016) when predicting state authenticity. Finally, we tested positive/negative affect and social desirability as alternative explanations for relationships between fit and authenticity. Data and analysis code are archived at https://osf.io/sb83d/?view_only=3d80718175b94914bdc6bf0a248f130a.

### Method

#### Participants

Participants were university students (*N* = 969; *M*_age_ = 21.89 years, *SD*_age_ = 4.91; 59.75% women, 37.25% men, 2.37% trans/nonbinary), who were predominately White (36.53%) or East Asian (21.67%). Study 1 involved two waves of data collection across three samples. Wave 1 included *n_SampleA_ =* 320 Canadian and *n_SampleB_ =* 219 American undergraduates. Wave 2 included a preregistered replication (AsPredicted #48026: https://aspredicted.org/UVB_YUR) of Wave 1 among *n_SampleC_ =* 540 American undergraduates recruited online via Prolific. Data collection continued until the semester (Wave 1) or funding (Wave 2) ended; participants received partial course credit (Wave 1) or $9.87 USD/hr (Wave 2). As results largely replicated across samples, we report analyses on a combined sample (results by Wave in Supplementary Materials). A sensitivity analysis in G*Power (Faul et al., 2009), conducted for a multiple regression model with three predictors, revealed that our final sample (*N =* 969) allowed us to detect a minimum standardized beta of β = .09, with 80% power, alpha = .05.

#### Procedure

Participants completed all measures clustered by scale, with scale order randomized. In addition to fit items, participants completed measures of state authenticity, dropout intentions, social belonging, autonomy, relatedness, competence, sense of self, goal motivation, positive/negative affect, socially desirable responding, and demographics. See Supplementary Materials for additional measures.

#### Measures

##### Three Types of Fit

Self-concept ( α = .94), goal ( α = .86), and social fit ( α = .89) were assessed with the university-framed items in Table 1.

##### State Authenticity

Participants rated their state authenticity on a single, face-valid item: “At [University], I feel. . .” (1 = *inauthentic*, 7 = *authentic*).^2^

##### Dropout Intentions

We assessed dropout intentions with four items created for this project. Three items were rated in terms of agreement (1 = *strongly disagree*, 6 = *strongly agree*): “I do not feel ‘emotionally attached’ to [University],” “I will likely actively look to transfer out of [University] in the next year,” and “I often think about dropping out” (1 = *strongly disagree*, 6 = *strongly agree*). A fourth item was rated in terms of frequency (1 = *never*, 5 = *always*): “This semester, how often have you thought about dropping out of school?” (1 = *never*, 5 = *always*). We reverse-scored and standardized responses before combining them into a composite ( α = .75).

##### Social Belonging

We assessed social belonging with a composite of three (reverse-scored) belonging uncertainty items (Yeager et al., 2016; e.g., “Sometimes I worry that I do not belong in college”; 1 = *not true at all*, 5 = *completely true*) and a fourth face-valid item (“I feel like I belong at [University]”; 1 = *strongly disagree*, 7 = *strongly agree; α* = .86).

##### Autonomy, Relatedness, and Competence

To assess SDT constructs, we used 21 items from the Basic Needs Satisfaction in General Scale (Johnston & Finney, 2010; 1 = *not at all true*, 7 = *very true*). Seven items referred to autonomy (e.g., “I feel like I am free to decide for myself how to live my life”; α _Autonomy_ = .75), eight to relatedness (e.g., “I really like the people I interact with”; α _Relatedness_ = .86), and six to competence (e.g., “People I know tell me I am good at what I do”; α _Competence_ = .74).

##### Sense of Self

We assessed sense of self with the 12-item Flury and Ickes (2007) Sense of Self Scale (SOSS; e.g., “I have a clear and definite sense of who I am and what I’m all about”; 1 = *very uncharacteristic of me*, 6 = *very characteristic of me*; α = .86).

##### Goal Motivation

To assess goal motivation, we had participants rate their most important goal with five statements (e.g., “I am motivated to pursue this goal”; 1 = *strongly disagree*, 7 = *strongly agree*; α = .87).

##### Positive and Negative Affect

We assessed positive and negative affect using the Scale of Positive and Negative Experience (SPANE; Diener et al., 2009). Participants read: “Please think about what you have been doing and experiencing since coming to [University],” and rated (1 = *very rarely or never*, 5 = *very often or always*) how frequently they experience six positive (e.g., “Good”; α _Positive_ = .90) and six negative (e.g., “Bad”; α _Negative_ = .85) varieties of affect.

##### Social Desirability

We measured social desirability using two subscales of the Balanced Inventory of Desirable Responding Short Form (BIDR-16—Hart et al., 2015; based on the BIDR-40, Paulhus, 1998): (a) self-deceptive enhancement (SDE, α = .72; e.g., “I am very confident of my judgments”), and (b) impression management (IM, α = .71; e.g., “I sometimes tell lies if I have to”). We analyzed the subscales separately given their modest correlation (*r =* .35, *p* < .001).

### Results

We provide descriptive statistics and variable intercorrelations in Table 2.

**Table 2.** Descriptive Statistics and Correlations Among Variables Measured in Study 1.

Variable	*M* (*SD*)	1	2	3	4	5	6	7	8	9	10	11	12	13	14	15
1. Self-concept fit (α = .94)	4.57 (1.47)															
2. Goal fit (α = .86)	4.87 (1.19)	.66***														
3. Social fit (α = .89)	4.49 (1.32)	.64***	.51***													
4. State authenticity (single item)	4.93 (1.39)	.61***	.53***	.61***												
5. Dropout intentions (α = .75)	0.00 (0.81)	−.62***	−.53***	−.43***	−.49***											
6. Social belonging (α = .86)	0.00 (0.84)	.58***	.55***	.52***	.54***	−.63***										
7. Autonomy (α = .75)	4.65 (0.91)	.40***	.42***	.49***	.46***	−.36***	.49***									
8. Relatedness (α = .86)	5.19 (1.00)	.41***	.36***	.50***	.42***	−.39***	.46***	.60***								
9. Competence (α = .74)	4.53 (1.05)	.38***	.43***	.38***	.43***	−.40***	.54***	.63***	.54***							
10. Positive affect (α = .90)	3.59 (0.70)	.63***	.56***	.50***	.54***	−.55***	.58***	.49***	.52***	.53***						
11. Negative affect (α = .85)	2.76 (0.76)	−.34***	−.43***	−.34***	−.36***	.37***	−.47***	−.43***	−.28***	−.43***	−.53***					
12. BIDR-16 (SDE) (α = .72)	3.81 (0.91)	.20***	.26***	.34***	.34***	−.23***	.41***	.44***	.27***	.48***	.34***	−.40***				
13. BIDR-16 (IM) (α = .71)	4.17 (0.95)	.14***	.19***	.26***	.25***	−.17***	.17***	.21***	.15***	.20***	.18***	−.22***	.35***			
14. Sense of self (α = .86)	3.79 (0.90)	.20***	.25***	.31***	.35***	−.27***	.46***	.54***	.42***	.59***	.34***	−.40***	.65***	.25***		
15. Goal motivation (α = .87)	6.02 (0.94)	.27***	.39***	.30***	.37***	−.29***	.36***	.43***	.36***	.49***	.42***	−.28***	.31***	.19***	.33***	

*Note.* We standardized dropout intentions and social belonging prior to forming composites, as response scales were different. BIDR = Balanced Inventory of Desirable Responding; SDE = Self-Deceptive Enhancement; IM = Impression Management.

* *p* < .05. **p < .01. ***p < .001.

#### Confirmatory Factor Analysis of Fit

**Figure 1.**
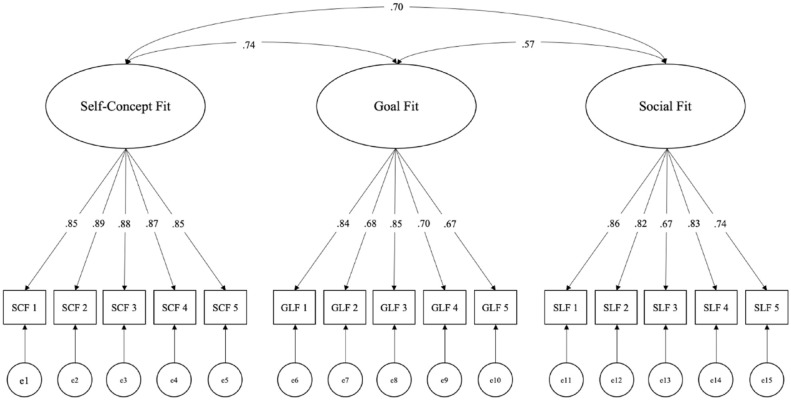
Confirmatory factor analysis of fit measures in Study 1.

Given that an exploratory factor analysis in the pilot study yielded a three-factor solution, we used confirmatory factor analysis (CFA) from the R package lavaan version 0.6-3 (Rosseel, 2012) to model each type of fit as an interrelated latent construct with five respective fit items and no residual correlations. We used full information maximum likelihood (FIML) estimation to account for missing data. The chi-square test of model fit was significant, χ ^2^ (87) = 362.80, *p* < .001, as is typical for large samples (Bentler & Bonett, 1980; Curran et al., 2003). Other fit indices, less biased by sample size, suggested good model fit; comparative fit index (CFI) = .97, root mean square error of approximation (RMSEA) = .06, standardized root mean squared residual (SRMR) = .04 (Clark & Watson, 2019; Hu & Bentler, 1999). Figure 1 depicts the full CFA model with factor loadings and covariances between latent constructs. All subscale items loaded ≥ .67 onto each latent factor.

The three fit factors were positively correlated, with self-concept and goal fit showing the strongest correlation (*r =* .74). However, a simplified two-factor model combining self-concept and goal fit items showed poorer fit to the data, χ ^2^(89) = 1198.77, *p* < .001, CFI = .89, RMSEA = .11, SRMR = .06, than the theoretically derived three-factor model, χ ^2^(87) = 362.80, *p* < .001, *p* < .001, CFI = .97, RMSEA = .06, SRMR = .04. Self-concept and social fit were also highly correlated (*r =* .70), and an alternative model combining the two also showed poorer fit to the data, χ ^2^(89) = 1537.58, *p* < .001, CFI = .86, RMSEA = .13, SRMR = .07, than the three-factor model. These results empirically support three distinct types of fit.

#### Effects of Fit on Students’ State Authenticity and Dropout Intentions

Having established three factors of the SAFE scale, we next conducted a structural regression model to examine the predictive effect of each type of fit on state authenticity (single-item measure). All three fit types uniquely predicted state authenticity: self-concept fit, β = .21, *p* < .001; goal fit, β = .20, *p* = < .001; social fit, β = .38, *p* < .001. The effect size for each relationship of fit-to-state authenticity was above the threshold ( β = .09) specified by the sensitivity analysis. Together, the three types of fit explained 49% of the variance in state authenticity, χ ^2^(99) = 383.80, *p* < .001; CFI = .97, Tucker-Lewis index (TLI) = .97, SRMR = .03, RMSEA = .06.

Next, a parallel structural regression model tested the predictive effect of each type of fit on dropout intentions, which was included in the model as a latent factor indicated by four observed items. Both self-concept fit, β = −.17, *p* = .007, and goal fit, β = −.41, *p* < .001, significantly and uniquely predicted dropout intentions. Social fit, β = −.01, *p* = .822, did not predict dropout intentions after accounting for the other types of fit. Together, this model explained 31% of the variance in latent dropout intentions with a good fit, χ ^2^(146) = 884.85, *p* < .001; CFI = .94, TLI = .93, SRMR = .08, RMSEA = .07.

**Figure 2.**
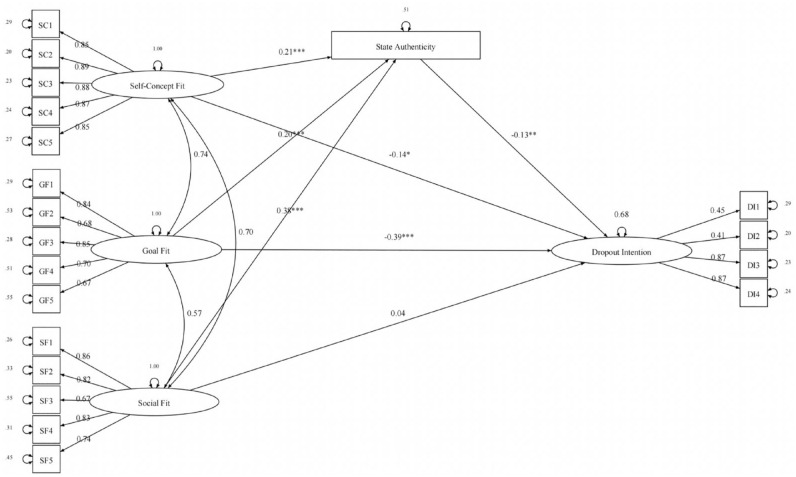
Relationship of each type of fit to organizational commitment as mediated via state authenticity in Study 1. *Note.* All coefficients are standardized. Regression coefficients are standardized; relationships among fit constructs reflect standardized covariances. Path coefficients are marked with *p < .05. **p < .01. ***p < .001.

Finally, a structural regression model tested state authenticity as a mediator of fit effects on latent dropout intentions, χ ^2^(161) = 906.492, *p* < .001; CFI = .94, TLI = .93, SRMR = .08, RMSEA = .07 (using the R package lavaan version 0.6-3, Rosseel, 2012; see Figure 2). All three types of fit uniquely predicted state authenticity: self-concept fit, β = .21, *p* < .001; goal fit, β = .20, *p* < .001; social fit, β = .38, *p* < .001. State authenticity predicted dropout intentions ( β = −.13, *p* = .002), and there were indirect effects of self-concept fit (a*b = −.03, *p* = .013), goal fit (a*b = −.03, *p* = .009), and social fit (a*b = −.05, *p* = .004). With state authenticity in the model, self-concept fit ( β = −.14, *p* = .024) and goal fit (*b* = −.39, *p* < .001) retained direct effects to dropout intentions, but not social fit ( β = .04, *p* = .424). See Table 3 for path coefficients and 95% confidence intervals.

**Table 3.** Structural Regression Modeling Results of State Authenticity Mediating Effects of Fit on Dropout Intentions.

Variable	State authenticity	Total effects of fit on dropout intentions	Direct effects of fit on dropout intentions	Indirect effects of fit on dropout intentions
Three types of fit-predicting outcomes				
Self-concept fit	β = .21*** [0.12, 0.31]	β = −.17** [−0.28, −0.05]	β = −.14* [−0.26, −0.02]	β = −.03* [−0.05, −0.01]
Goal fit	β = .20*** [0.11, 0.28]	β = −.41*** [−0.51, − 0.31]	β = −.39*** [−0.49, −0.29]	β = −.03** [−0.05, −0.01]
Social fit	β = .38*** [0.31, 0.46]	β = −.01 [−0.10, 0.08]	β = .04 [−0.06, 0.14]	β = −.05** [−0.09, −0.02]
State authenticity predicting dropout intentions				
β = −.13** [−0.22, −0.05]				

*Note.* The betas represent standardized coefficients, with their 95% confidence intervals.

* *p* < .05. **p < .01. ***p < .001.

#### Tests of Convergent and Discriminant Validity

Next, we tested the convergent and discriminant validity of the SAFE scale by examining the relationship of fit to other, conceptually similar but distinct measures.

##### Convergent Validity With Belonging

In the aggregate, the SAFE scale aims to assess a construct similar to—but more nuanced than—belonging, by differentiating among different types of fit. Thus, two regression models predicted belonging from: (a) a composite of all three types of fit, and (b) the three types of fit separately. As expected, the composite of all three fit types was strongly related to belonging, β = .63, *p* < .001, yielding evidence of convergent validity. In addition, when entered simultaneously, each fit type had a unique relationship with belonging (self-concept fit, β = .27, *p* < .001; goal fit, β = .25, *p* < .001; social fit, β = .21, *p* < .001), all with effects above the threshold ( β = .09) specified by sensitivity analyses.

##### Discriminant Validity From Other Distinct Constructs

To assess the discriminant validity of the SAFE scale, we tested two separate models. Model 1 predicted state authenticity from self-concept, goal, and social fit while controlling for SDT constructs (autonomy, relatedness, competence). Model 2 accounted for sense of self, goal motivation, and social belonging (conceptually related to self-concept, goal, and social fit, respectively).^3^ As shown in Table 4, all three fit types predicted unique variance in state authenticity beyond other similar but distinct constructs, establishing discriminant validity.

**Table 4.** Results of Models Testing Divergent Validity by Controlling for Conceptually Related Variables When Regressing State Authenticity Onto Fit Measures in the Combined Analysis in Study 1.

	Original model	Self-determination theory	Motivation, self, and belonging constructs	Positive and negative affect	Socially desirable responding constructs
Variable	Model 0	Model 1	Model 2	Model 3	Model 4
Self-concept fit	.27*** [0.20, 0.35]	.26*** [0.19, 0.33]	.27*** [0.19, 0.34]	.21*** [0.13, 0.29]	.31*** [0.24, 0.38]
Goal fit	.17*** [0.10, 0.23]	.12*** [0.05, 0.19]	.07* [0.00, 0.14]	.12*** [0.05, 0.18]	.13*** [0.06, 0.19]
Social fit	.34*** [0.28, 0.41]	.29*** [0.22, 0.35]	.26*** [0.19, 0.32]	.32*** [0.25, 0.38]	.28*** [0.22, 0.35]
Competence	—	.11*** [0.05, 0.17]	—	—	—
Autonomy	—	.08* [0.01, 0.15]	—	—	—
Relatedness	—	.02 [−0.05, 0.08]	—	—	—
Sense of self	—	—	.12*** [0.06, 0.17]	—	—
Goal motivation	—	—	.12*** [0.06, 0.17]	—	—
Belonging	—	—	.11*** [0.05, 0.18]	—	—
Positive affect	—	—	—	.16*** [0.09, 0.23]	—
Negative affect	—	—	—	−.04 [−0.10, 0.01]	—
BIDR (SDE)	—	—	—	—	.13*** [0.07, 0.18]
BIDR (IM)	—	—	—	—	.07** [0.02, 0.12]

*Note.* The reported coefficients in this table are standardized coefficients, with 95% confidence intervals. BIDR = Balanced Inventory of Desirable Responding; SDE = Self-Deceptive Enhancement; IM = Impression Management.

* *p* < .05. **p < .01. ***p < .001.

#### Ruling Out Alternative Explanations

Finally, to test whether the relationships between fit and state authenticity were better explained by positive and negative affect or social desirability, we tested two additional models predicting state authenticity from each fit type and each set of control variables (Model 3 included positive/negative affect and Model 4 included social desirability; Table 4). Positive affect had the strongest relationship to state authenticity, supporting evidence that state authenticity is experienced positively (Lenton, Slabu, et al., 2013). However, each fit type predicted unique variance in state authenticity even when accounting for alternative explanatory variables.

### Discussion

Study 1 provided empirical support for three types of fit that can be independently measured to uniquely predict state authenticity. Although the three fit types can be distinct routes to state authenticity, they need not all predict any given outcome. Indeed, students’ self-concept and goal fit, but not social fit, directly predicted their dropout intentions. That said, all three fit types showed significant indirect effects on dropout intentions via state authenticity. Importantly, the SAFE scale had a good factor structure, showing convergent and discriminant validity from other related constructs, and being robust to alternative explanations in relation to authenticity (i.e., positive/negative affect, social desirability).

Although Study 1 validated our tripartite conceptualization of fit, the cross-sectional survey methodology only tested between-person (not between-situation) variation in responses. As such, our findings yielded evidence of fit and authenticity as situated within a context. Study 2 aimed to assess momentary variation in experiences of fit, authenticity, and relevant outcomes.

## Study 2

In Study 2, we used an experience sampling design to isolate within-person effects (as distinct from between-person effects) of fit and authenticity as linked to variation in contextual cues and outcomes (see Supplementary Materials). We archived materials (i.e., data, analysis code, preregistration) at https://osf.io/s9y85/?view_only=fb98a7ae10564c0fbc93a0bd1dcd214e. Our primary hypothesis was that in university situations where students feel greater self-concept fit, goal fit, and/or social fit, they will also report greater authenticity, willingness to return to the situation, and state attachment to their university. To establish the implications of misfit and inauthenticity beyond self-report measures, Study 2 also included a performance-based measure of working memory capacity. Reduced working memory capacity is often linked to burnout (Gavelin et al., 2022), which we also measured, and thus linking these outcomes to experiences of misfit could provide important evidence of cognitive disfluency. We preregistered analyses to test the unique role of each type of fit in predicting all outcomes, with no specific hypotheses about the relative strength of these relationships.

The experience sampling design allowed us to isolate how each type of fit is uniquely cued by contextual features. Drawing from the SAFE model, we preregistered hypotheses that students would experience: (a) higher self-concept fit in situations that were familiar or freely chosen, as these situations would activate the default self-concept, (b) higher goal fit in situations involving active (vs. passive) engagement or social (vs. solitary) actions, as these situations would afford valued goals, and (c) higher social fit in presence of close (vs. non-close) others, as these situations would foster social validation.

### Method

#### Procedure

Study 2 was embedded within a larger longitudinal project with three phases: a T1 baseline survey about one month after the start of the term, a two-week experience sampling phase, and a T2 survey at the end of the term (T1 and T2 surveys are less relevant to the current study; see Supplementary Materials). Participants began the 14-day experience sampling phase of the study approximately six to eight weeks after the term started. Each day, participants received emailed survey prompts at 2 p.m., 5 p.m., and 8 p.m. The three-minute survey was accessible via laptop, smartphone or tablet and contained questions about the current context (e.g., “What are you doing right now?”), state authenticity, and fit, as well as momentary outcomes (i.e., likelihood to return, state university attachment, burnout, working memory capacity).

#### Participants

Our preregistered sample size was 220, based on Monte Carlo simulations (Arend & Schäfer, 2019), but we continued to recruit participants for two academic terms at a Canadian University and compensated them with research credit in psychology classes or payment. Of the 290 students who enrolled in the study, 37 were excluded from analyses (as preregistered) because they completed no experience sampling surveys about on-campus experiences. In this final sample of 253 (54.15% first-year undergraduates, 45.85% second-year undergraduates; *M*_age_ = 18.98 years, *SD*_age_ = 1.76; 80.24% women, 16.60% men, 2.37% non-binary, 0.39% non-specified gender; East Asian: 39.13%, White/European:18.58%, South Asian: 17.00%, Southeast Asian: 5.93%, each other ethnicity < 5.00%), 252 completed more than 50% questions in T1 survey. Each of these 253 participants completed an average of 9.68 experience sampling surveys on campus, *SD* = 8.04, *Min* = 1, *Max* = 41. Seventeen participants completed all 42 survey prompts (including both on-campus and off-campus experiences),^4^ and 140 participants completed more than 80%, qualifying them for a $5 bonus. At the end of the academic term, 67.19% of 253 participants completed the T2 survey.

#### Experience Sampling Measures

##### Contextual Information

We measured location with a single question: “Where are you right now?” and five response options (“*at home*,” “*familiar place on campus*,” “*unfamiliar place on campus*,” “*familiar place off-campus*,” and “*unfamiliar place off-campus*”). We also asked participants, “Did you choose to be here?” (yes/no) and “What are you doing right now?” (“*doing something active* [*e.g., studying, exercising, working*],” “*doing something passive* [*e.g., watching TV, reading, browsing the web, relaxing*],” “*doing something social* [*e.g., talking with friends or family*],” “*doing something solitary* [*e.g., staying by yourself*”]). To assess social company, participants responded to the question stem: “I am with. . .” (“*solo: I’m alone*,” “*close others: friends/relationship partner/family*,” “*non-close others: acquaintances(classmates/coworkers)/strangers*.” Note that company categories were not mutually exclusive; participants could be with both close and non-close others.

##### Momentary Fit

We assessed each type of fit with the highest-loading item from their respective subscale in Study 1, Wave 1: self-concept fit (“Just being here in this space suits the way I see myself”), goal fit (“This is a place where I feel intrinsically motivated by my own goals”), and social fit (“I can act natural around the people who are here”) on a scale ranging from 1 (*strongly disagree*) to 7 (*strongly agree*).

##### State Authenticity

Participants rated their state authenticity at their university on a single item: “At [University] I feel. . .” (1 = *inauthentic*, 7 = *authentic*).

##### Behavioral Intentions

Participants rated, “Are you likely to return to this setting?” (1 = *definitely not*, 7 = *definitely yes*).

##### State Attachment

Participants reported their state attachment with their university with a single rating (1 = *completely emotionally disengaged from [University]*, 7 = *strongly emotionally attached to [University]*).

##### Burnout

Participants reported their burnout on the item, “Right now, I feel emotionally drained” (1 = *none at all*, 7 = *extremely*).

##### Working Memory Capacity

We added a measure of working memory capacity to represent a performance-based measure of cognitive fluency. To avoid cognitive overload, we only included the measure in 50% of the experience sampling prompts (randomly selected). On these occasions, after filling out self-report measures, participants completed a memory updating task that correlates highly with other working memory measures (Oberauer et al., 2000) and has been used in previous experience sampling research (Riediger et al., 2011). We trained participants on the memory updating task during the T1 survey. The memory updating task started by presenting participants with a 2 × 2 matrix of frames (four frames in total; see Supplementary Materials). Four single-digit numbers (one per frame) were displayed simultaneously for 6.5 seconds, and participants were instructed to memorize the four numbers. The four numbers then disappeared. A single-digit addition or subtraction updating operation (e.g., +4) appeared in one of the frames. Participants’ task was to update the original number in the corresponding frame according to the operation and hold that new number in working memory. After 3.5 seconds, the updating operation disappeared, and a new operation was presented in a different frame. Participants completed four updating operations, requiring them to remember four updated numbers that, in the end, were asked to input into a blank 2 × 2 matrix.

The working memory task was programmed in Qualtrics to only accept numeric responses and R code double validated that. For each working memory trial, we calculated an accuracy score as the proportion of numbers answered correctly, with possible scores being 0.00, 0.25, 0.50, 0.75, and 1.00. Initial data screening revealed that only 10 participants received 0.00 correct responses on all of their trials (constituting only 19, or less than 2%, of the 1,197 working memory trials collected). These scores were retained and thus no observations on this measure were excluded.

#### Dispositional Authenticity

The T1 survey included a 12-item measure of dispositional authenticity (Wood et al., 2008; e.g., “I think it is better to be yourself, than to be popular”; 1 = *does not describe me at all*, 7 = *describes me very well*; α = .82).

### Results

#### Analysis Plan

We used multilevel modeling (R package ‘lme4’; Bates et al., 2015) with each short survey response as a level-1 unit and each person as a level-2 cluster. We cluster-mean centered all continuous level-1 predictors (momentary ratings of the three fit types) to disaggregate within-person and between-person effects, and we grand-mean centered any level-2 predictors (e.g., dispositional authenticity; Raudenbush & Bryk, 2002; Rights & Sterba, 2019). As preregistered, we analyzed only on-campus experiences (2,448 out of 8,222 total observations) and did not control for the day of a week.

Table 5 summarizes descriptive statistics and bivariate correlations (for both within-person and between-person levels). Correlations among the three fit types and state authenticity were positive, but the magnitude of relationships was smaller at the within-person than between-person level. Dispositional authenticity was modestly correlated with between-person variance in fit and authenticity but uncorrelated with within-person variance in these measures. These patterns support distinguishing these constructs at the state level.

**Table 5.** Descriptive Statistics and Correlations Among Key Variables in Study 2.

Variable	*M* (*SD*)	1	2	3	4	5	6	7	8	9
1. Self-concept fit	5.21 (1.22)		.82***	.73***	.62***	.56***	.49***	−.12***	.10***	.31***
2. Goal fit	5.14 (1.36)	.43***		.60***	.59***	.46***	.52***	−.15***	.13***	.23***
3. Social fit	5.20 (1.46)	.33***	.19***		.60***	.49***	.43***	−.07***	.05*	.33***
4. State authenticity	4.99 (1.30)	.27***	.3`***	.25***		.36***	.85***	−.15***	−.01	.33***
5. Likelihood to return	5.73 (1.40)	.28***	.23***	.26***	.17***		.23***	−.05*	.17***	.21***
6. State attachment	4.76 (1.45)	.22***	.26***	.11***	.38***	.10***		−.12***	.05*	.22***
7. Burnout	3.97 (1.69)	−.15***	−.05	−.11***	−.16***	−.05	−.18***		−.09***	−.11***
8. Working memory capacity	0.76 (0.32)	.06	.01	−.06*	−.02	.02	−.01	−.02		−.05*
9. Dispositional authenticity (α = .82)	4.60 (0.89)	−.01	.01	.00	−.00	−.00	.01	.01	−.00	

*Note.* Variables (1) to (8) are single-item measures. The means and *SD*s we present here are the grand-means and *SD*s. Correlations above the diagonal are between-person, whereas those below the diagonal are within-person, except for the last row showing level-1 to level-2 correlations.

* *p* < .05. **p < .01. ***p < .001.

Among on-campus observations, 86.85% of participants were at familiar places and had chosen to be there in 94.04% of the cases. Participants were often engaged in something active (58.37%, vs. passive), and 21.24% of the time they were actively engaged in social (vs. solitary) activity. On 36.73% of occasions, participants reported that they were alone, on 40.44% only with close others, on 15.43% only with non-close others, and on 7.40% with both close and non-close others.

#### Does Momentary Variation in Fit Predict State Authenticity?

Using multilevel modeling with random intercepts and slopes, we regressed state authenticity onto both within-person and between-person components of all three fit measures, allowing the intercept and slopes to vary across individuals. Supporting preregistered hypotheses, all three fit types significantly predicted state authenticity within-person: self-concept fit, β = .11, *p* < .001; goal fit, β = .12, *p* < .001; social fit, β = .14, *p* < .001. Together, the three types of fit explained 9.07% of the total variance^5^ (computed with Rights & Sterba’s, 2019, procedure). Thus, when participants reported higher levels of each type of fit, they also reported feeling more authentic.

The between-person level analyses replicated patterns from Study 1. Participants who, on average across situations, reported higher state authenticity also reported, on average, higher self-concept fit, β = .20, *p* = .03, 95% CI [.02, .39]; goal fit, β = .24, *p* = .006, 95% CI [0.07, 0.41]; and social fit, β = .48, *p* < .001, 95% CI [0.35, 0.62]. Thus, at the between-person level, the three types of fit also explained the total variance in authenticity, *R*^2^ = .34.^6^

#### Does Momentary Variation in Fit Predict Momentary Outcomes?

Next, we regressed each outcome variable on both within-person and between-person components of all three types of fit, allowing the intercept and slope to vary across participants in multilevel modeling. We summarize results for within-person effects in Table 6, as these are the primary focus of the study. These findings control for between-person effects, which are reported in Supplementary Materials.

**Table 6.** Within-Person Results of Momentary Fit and Authenticity Predicting Momentary Outcomes.

Variable	State authenticity	Willingness to return	State attachment	Working memory	Burnout
Predicting outcomes from fit					
Momentary self-concept fit	β = .11*** [0.07, 0.16]	β = .16*** [0.10, 0.21]	β = .10*** [0.06, 0.15]	β = .12** [0.04, 0.19]	β = −.12*** [−0.18, −0.06]
Momentary goal fit	β = .12*** [0.07, 0.16]	β = .11*** [0.06, 0.16]	β = .13*** [0.08, 0.17]	β = −.04 [−0.10, 0.03]	β = −.03 [−0.09, 0.02]
Momentary social fit	β = .14*** [0.10, 0.19]	β = .15*** [0.10, 0.20]	β = .07** [0.02, 0.11]	β = −.08* [−0.16, −0.01]	β = −.04 [−0.10, 0.02]
Predicting outcomes from state authenticity					
State authenticity	NA	β = .23*** [0.15, 0.31]	β = .36*** [0.30, 0.43]	β = −.01 [−0.10, 0.08]	β = −.23*** [−0.30, −0.15]

*Note.* 95% confidence intervals are reported in brackets. Variables (1) to (8) are single-item measures. We conducted separate models to test the unique predictive effects of fit (in one model to predict state authenticity and another set of models to predict outcomes) and state authenticity (in a separate model).

* *p* < .05. **p < .01. ***p < .001.

##### State Attachment to the University

The within-person components of self-concept fit ( β = .10, *p* < .001), goal fit ( β = .13, *p* < .001), and social fit ( β = .07, *p* = .005) each uniquely and significantly predicted participants’ state attachment to the university and explained 7.46% of the total variance in this variable. Although in Study 1 social fit was not directly related to dropout intentions, in Study 2 it did uniquely predict state attachment.

##### Willingness to Return to the Situation

Supporting preregistered hypotheses, in those situations where participants felt more self-concept fit, β = .16, *p* < .001, goal fit, β = .11, *p* < .001, and/or social fit, β = .15, *p* < .001, they also reported significantly higher willingness to return to that situation. Taken together, the within-person components of the three types of fit explained 13.17% of the total variance in behavioral intentions.

##### Burnout

Analysis of burnout revealed a different pattern. Participants reported greater burnout in situations where they experienced less self-concept fit, β = −.12, *p* < .001, explaining 3.73% of the total variance. Neither goal fit nor social fit significantly predicted burnout.

##### Working Memory Capacity

Similarly, participants’ working memory capacity was higher in situations where they felt higher self-concept fit ( β = .12, *p* = .004), but also lower social fit ( β = −.08, *p* = .020), explaining 1.64% of the total variance. Goal fit did not significantly predict momentary variation in working memory capacity, and none of the between-person components of the three types of fit significantly predicted working memory capacity (see Supplementary Materials), revealing the contextualized nature of this outcome.

#### Does Momentary Variation in Authenticity Predict Momentary Outcomes?

Having established that each outcome was uniquely predicted by one or more measures of fit, we next predicted each outcome from state authenticity (in place of momentary fit). As hypothesized, in situations where participants felt more authentic, they reported a higher willingness to return, β = .23, *p* < .001, higher state attachment to the university, β = .36, *p* < .001, and lower burnout, β = −.23, *p* < .001. However, state authenticity did not predict momentary variation in working memory capacity, β = −.01, *p* = .79.

**Figure 3.**
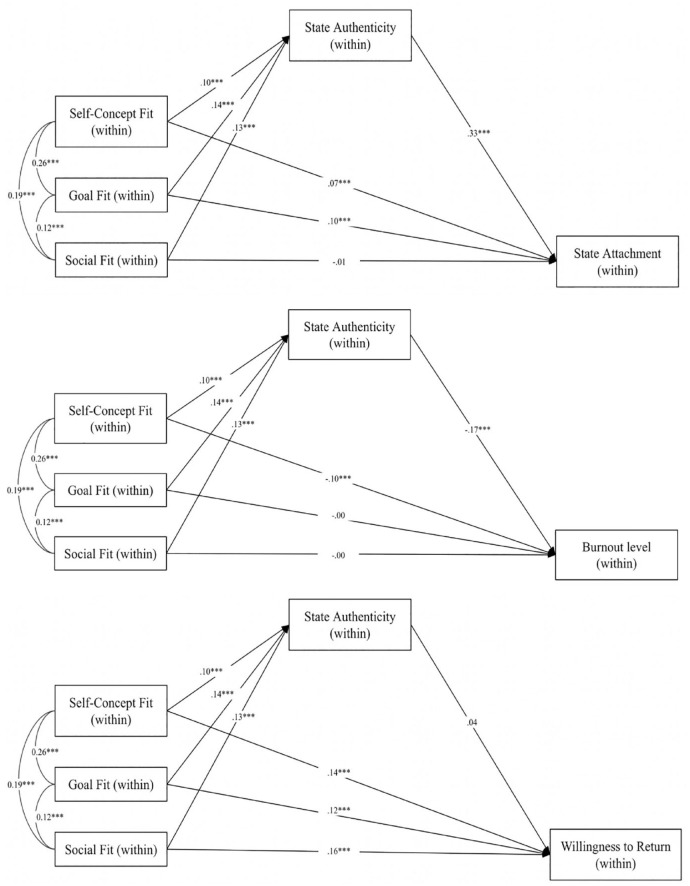
Within-person relationship of each type of fit to state attachment to the university, burnout, and willingness to return as mediated through state authenticity in the combined analysis. *Note.* Path coefficients reflect standardized betas; relationships among fit constructs reflect raw covariances (i.e., estimates may surpass 1.00).

As in Study 1, we conducted path analysis with multilevel data structure using R package lavaan version 0.6-3 (Rosseel, 2012) and focused on within-person variance. Given the lack of a significant relationship between within-person state authenticity and working memory capacity, we only conducted these exploratory analyses involving willingness to return, state attachment, and burnout. Each type of fit showed significant indirect effects on momentary state attachment to the university: self-concept fit a*b = .03, *p* < .001, 95% CI [0.02, 0.05], goal fit a*b = .05, *p* < .001, 95% CI [0.03, 0.06], social fit a*b = .04, *p* < .001, 95% CI [0.03, 0.06]. For burnout, the direct effect between self-concept fit on burnout was mediated by state authenticity (indirect effect: a*b = −.02, *p* < .001, 95% CI [−0.03, −0.01]). However, the effects of fit on willingness to return were unmediated by state authenticity (Figure 3).

#### Do Features of the Context Uniquely Predict Different Types of Fit?

Finally, we tested preregistered hypotheses about the types of contextual features that predict each type of fit. We ran a series of multilevel models with random intercepts regressing a given momentary fit rating (controlling for other fit measures) on each contextual variable (see Table 7; company was represented with three dummy-coded variables: “only close others,” “only non-close others,” and “both close and non-close others;” with “alone” as the reference group).

**Table 7.** Contextual Features Predicting Momentary Fit.

	Self-concept fit		Goal fit		Social fit	
Variable	Not controlling for other fit	Controlling for other fit	Not controlling for other fit	Controlling for other fit	Not controlling for other fit	Controlling for other fit
Choose to be here	0.56*** [0.40, 0.71]	0.31*** [0.18, 0.44]	0.38*** [0.21, 0.54]	0.07 [−0.07, 0.21]	0.36*** [0.21, 0.51]	0.13 [−0.01, 0.28]
Familiar place	0.29*** [0.19, 0.39]	0.11* [0.02, 0.19]	0.24*** [0.13, 0.35]	0.07 [−0.03, 0.16]	0.32*** [0.22, 0.42]	0.21*** [0.12, 0.30]
Actively engaged	0.13** [0.04, 0.21]	−0.01 [−0.08, 0.06]	0.41*** [0.32, 0.49]	0.36*** [0.28, 0.43]	−0.12** [−0.20, −0.04]	−0.22*** [−0.30, −0.14]
Social activities	0.23** [0.09, 0.37]	0.07 [−0.05, 0.18]	0.13 [−0.03, 0.28]	0.01 [−0.13, 0.15]	0.29*** [0.15, 0.43]	0.19** [0.06, 0.31]
With only close others	0.10* [0.02, 0.19]	0.004 [−0.07, 0.08]	0.01 [−0.08, 0.10]	−0.07 [−0.15, 0.01]	0.32*** [0.24, 0.40]	0.29*** [0.21, 0.36]
With only non-close others	−0.22*** [−0.33, −0.11]	−0.02 [−0.11, 0.07]	−0.11 [−0.22, 0.01]	0.05 [−0.06, 0.15]	−0.50*** [−0.61, −0.40]	−0.43*** [−0.53, −0.34]
With both close and non-close others	−0.04 [−0.19, 0.10]	0.08 [−0.04, 0.20]	−0.15^†^ [−0.31, 0.00]	−0.13^†^ [−0.26, 0.01]	−0.15* [−0.29, −0.01]	−0.12^†^ [−0.25, 0.01]

*Note.* 95% confidence intervals are reported in brackets.

† *p* < .10. *p < .05. **p < .01. ***p < .001.

##### Self-Concept Fit

Supporting preregistered hypotheses, participants experienced more self-concept fit in situations that were familiar (vs. unfamiliar), β = .11, *p* = .01, or freely chosen, β = .31, *p* < .001.^7^ No other contextual variable predicted self-concept fit uniquely.

##### Goal Fit

Partially supporting hypotheses, participants experienced greater goal fit when engaged in active (vs. passive) activities, β = .36, *p* < .001, but not when engaged in social (vs. solitary) activity, β = .01, *p* = .89. No other contextual variable predicted goal fit uniquely.

##### Social Fit

As hypothesized, participants experienced significantly greater social fit in situations with only close others (vs. alone), β = .29, *p* < .001, and significantly less social fit in situations with only non-close others (vs. alone), β = −.43, *p* < .001. Although not preregistered, participants also experienced greater social fit in situations that were familiar, and unsurprisingly, when engaged in social (vs. solitary) activities. They also experienced less social fit during activities that were active versus passive (Table 7).

### Discussion

Study 2 confirmed that three types of fit uniquely predict state authenticity and other momentary outcomes. Although results of between-person analyses constitute a conceptual replication of Study 1, the experience sampling method provides greater insight into how fit and authenticity vary from one situation to the next within-person. Controlling for individual differences, perceiving one’s environment as a fit to one’s self-concept, goals, and sociality offers independent pathways to feeling authentic in the moment. That said, momentary variation in fit only explained 9.07% of the total variation in state authenticity, suggesting that other unmeasured variables (both within and between-person) also play a role.

In line with the SAFE model, three distinct types of fit are predictive of situation selection, measured as one’s willingness to return to the situation. Just as fit and authenticity predicted students’ dropout intentions in Study 1, in Study 2 momentary variation in each type of fit (including social fit) predicted students’ state attachment to their university. Moreover, consistent with the SAFE model’s proposition that self-concept fit cues cognitive fluency, only self-concept fit predicted higher working memory capacity and lower burnout. Unexpectedly, social fit also predicted lower working memory capacity, perhaps because people were more distracted around close others. A puzzle of Study 2 is that state authenticity did not statistically mediate the effects of fit on one’s willingness to return to the situation, though there was evidence consistent with mediation for fit effects on state attachment and burnout (especially for self-concept fit). We revisit this issue in the General Discussion.

Finally, evidence supported preregistered hypotheses about contextual features that predict each type of fit. Choosing to be in a familiar place elicits self-concept fit, whereas active (vs. passive) engagement in a situation elicits goal fit. Social fit is elicited in a wider range of contexts: when people are with close (vs. non-close) others, in familiar places, and passively engaged in shared activities. Taken together, these patterns provide contextual evidence that these types of fit represent conceptually distinct ways in which individuals experience state authenticity as fit to their environment.

## General Discussion

The present research tested key tenets of the SAFE model (Schmader & Sedikides, 2018), which posits three distinct types of person-environment fit—self-concept, goal, and social—that predict state authenticity and one’s attraction to, or attrition from, a given context. We tested these hypotheses across two studies examining university students’ fit and authenticity on campus, given the possible consequences of these experiences for students’ engagement. Our work has theoretical, methodological, and practical implications.

Theoretically, the findings provide the first empirical support of key hypotheses derived from the SAFE model. Across multiple samples and two different methods, students reported greater authenticity to the degree that the context afforded higher self-concept, goal, and/or social fit. We established the predictive validity of a new SAFE scale using between-person analyses of students’ fit and authenticity felt at their university (Study 1) and within-person analyses of three fit types across specific situations on campus (Study 2). These analyses confirm that although each type of fit is related to one another, they are also distinct predictors of state authenticity.

Furthermore, tests of convergent validity indicated that an undifferentiated measure of belonging is related to each type of fit, suggesting that research on belonging might be enhanced by distinguishing fit stemming from social acceptance from fit stemming from passive cues to the default self or from active engagement with valued goals. Tests of discriminant validity show that, although the SAFE scale is correlated with other related constructs (autonomy, relatedness, competence, sense of self, goal motivation, and belonging), it explains variability in authenticity that is unique from these variables. Finally, each fit’s relationship to state authenticity cannot be fully explained by positive affect or social desirability.

Study 2 provided evidence of the environmental cues that elicit each type of fit. Goal fit was uniquely experienced during active (vs. passive) activities, whereas self-concept fit was uniquely experienced in familiar and chosen situations, and social fit was uniquely experienced when with close (and without non-close) others. These results further reveal that each type of fit provides a unique pathway to state authenticity.

In addition to these theoretical advances, we psychometrically validated a new multidimensional SAFE scale that can be adapted to a variety of contexts. A pilot study provided initial evidence for a three-factor model corresponding to the three types of fit in the SAFE model. In Study 1, a CFA showed that our predicted three-factor model performed better than two-factor alternatives and yielded reliable scales. Single-item measures of each fit were related to distinct contextual factors in Study 2’s experience sampling paradigm. Thus, our studies provide researchers with easy-to-administer measures for examining distinct types of fit.

Our work makes a practical advance by linking fit and state authenticity to meaningful student outcomes. In Study 1, this included evidence that self-concept and goal fit (but not social fit) uniquely predicted students’ dropout intentions, as statistically mediated by state authenticity. In Study 2, within-person analyses revealed that momentary variation in each fit type uniquely predicted not only state authenticity but also willingness to return to the situation and state attachment to one’s university. Such findings could inform interventions to enhance different types of fit. For example, if students report leaving a university computer science program because of social misfit, interventions might focus on facilitating social inclusion for those students. Low self-concept fit would instead suggest interventions that cue a sense of familiarity inclusive of students with a diversity of backgrounds or interests.

Although the present research focused on fit and authenticity in a general sample, the SAFE model provides a framework to examine how individuals with devalued social identities self-select out of domains where they systematically feel a lack of fit and authenticity. Ancillary analyses in Study 1 revealed that White students scored significantly higher than non-White students on self-concept, goal fit, and state authenticity (Supplementary Materials). Study 2’s smaller sample size made similar tests underpowered although mean comparisons between White and non-White students were in the same expected direction. As such, our findings complement research on cultural (mis)match and belonging (Stephens et al., 2012), self-esteem (Fulmer et al., 2010), and well-being (Götz et al., 2018). Having validated our multidimensional SAFE scale across several broad samples of university students, future work is needed to examine the effects of marginalization on these different types of fit, with possible implications for disparities in motivation, performance, and/or attrition and tailored interventions to close those gaps.

### Limitations and Future Directions

The correlational design of this research precludes causal inferences about the relationship between fit and state authenticity or between state authenticity and engagement with the environment. However, the experience sampling methodology of Study 2 allows us to link these responses to ecologically valid features of the environment and approach/avoidance intentions. Causal relationships can be established in future experimental research that independently manipulates each type of fit to observe effects on other components of the SAFE model. Assuming that fit causally boosts engagement via state authenticity, there may also be recursive processes at work: feeling higher state authenticity might increase engagement and reinforce one’s fit to the environment.

Although our findings relied mainly on self-report measures, we note that subjective sense of fit and authenticity are critical for situational engagement. In addition, Study 2 included a performance measure of working memory capacity and revealed that cognitive fluency was enhanced in situations that afforded high self-concept (and low social) fit. This link between self-concept fit and working memory capacity is consistent with the theorized link between self-concept fit and cognitive fluency (Schmader & Sedikides, 2018). Future research could include behavioral measures of motivational fluency elicited by goal fit (e.g., task persistence) and interpersonal fluency elicited by social fit (e.g., speech hesitations), as well as objective measures of situational selection or avoidance.

Finally, although we focused on intrapersonal experiences of state authenticity, state authenticity can also operate interpersonally, vis-à-vis the relational self (Chen, 2019; Chen et al., 2006). Interpersonal experiences of state authenticity might vary, as relational authenticity is predicted by enacting an ideal, as opposed to an actual, self. Future investigations might also examine how state authenticity is expressed much like nonverbal emotional expression. Expressions of state authenticity might communicate norms about who will fit in that setting.

### Conclusion

Feelings of fit and belonging have long been considered by laypeople and social scientists alike as driving decisions about which environments people enter or exit. The SAFE model extends this literature by delineating the different ways in which people feel a sense of fit to their environment (self-concept, goal, and social) and how these feelings of fit predict situation selection via state authenticity. By empirically distinguishing these three types of fit, our work provides a theoretical framework and newly validated measures to guide research on when, why, and how people self-select into some situations and out of others.

## Supplemental Material

sj-docx-1-psp-10.1177_01461672231223597 – Supplemental material for The SAFE Model: State Authenticity as a Function of Three Types of Fit Supplemental material, sj-docx-1-psp-10.1177_01461672231223597 for The SAFE Model: State Authenticity as a Function of Three Types of Fit by Audrey Aday, Yingchi Guo, Smriti Mehta, Serena Chen, William Hall, Friedrich M. Götz, Constantine Sedikides and Toni Schmader in Personality and Social Psychology Bulletin

## Notes

1. Although self-concept and goal fit correlated .70, in this pilot study as in Study 1, a three-factor model provided a much greater fit to the data than a two-factor model (see SOM for details).

2. A second visual measure of authenticity (Real-Self Overlap Scale; Lenton, Bruder, et al., 2013) was positively related to the first, *r* = .60, *p* < .001, and effects relying on a combined measure were similar (Supplementary Materials). We report results only on the face valid measure to minimize construct overlap with fit measures.

3. We included social belonging in tests of both convergent and divergent validity because in past work (Yeager et al., 2016), belonging has been defined in terms of social acceptance but measured with items that more broadly capture fit. We thus tested the assumption that the SAFE scale is convergent to this commonly used measure that references fit; but that our social fit scale is divergent from this as a measure of social belonging.

4. Three participants completed over 42 valid surveys due to their continuous reporting until T2.

5. The within-person variation of three types of fit accounted for 31.08% of the within-person variance in state authenticity.

6. All results are unchanged when controlling for dispositional authenticity measured in the T1 survey (Supplementary Materials).

7. A restriction in range on the chosen variable might constrain the size of this effect.
