# Supplementary material for: The SAFE Model: State Authenticity as a Function of Three Types of Fit
Source: Pers Soc Psychol Bull. 2024 Jan 28;51(8):1472–89. doi: 10.1177/01461672231223597 (PMC12206244; doi:10.1177/01461672231223597)
Supplement: sj-docx-1-psp-10.1177_01461672231223597 – Supplemental material for The SAFE Model: State Authenticity as a Function of Three Types of Fit [file sj-docx-1-psp-10.1177_01461672231223597.docx]

**Supplementary Materials**

**The SAFE Model: State Authenticity as a Function of Three Types of Fit**

*Table of Contents*

| Section | Page |
| --- | --- |
| Pilot Study – Summary of Key Measures | 1 |
| Pilot Study - Results  Study 1 – Supplemental Materials | 4  11 |
| Study 1 – Supplemental Results: Factor Analysis | 17 |
| Study 1 – Supplemental Results: Core Findings | 22 |
| Study 1 – Comparisons by Race/Ethnicity | 26 |
| Study 2 – Supplemental Measures | 27 |
| Study 2 – Supplemental Results | 29 |

**Pilot Study**

Our goal for this pilot study was to test whether state authenticity and commitment to one’s organization are a function of three distinct types of fit. To this end, we developed a fit measure containing three distinct subscales to assess self-concept, goal, and social fit. We internally generated an initial pool of items drawing on theoretical considerations from the SAFE model, all framed within an organizational context (see Table S2 for item wording). We then administered these items to a sample of working adults and subjected all three subscales to an exploratory factor analysis (EFA) to determine whether a three-factor solution corresponding to self-concept, goal, and social fit emerged. Using path modeling, we tested our theoretically- derived hypotheses that each type of fit would explain unique variability in participants’ state authenticity, which, in turn, would statistically mediate effects on their organizational commitment (i.e., commitment to their current job).

**Method**

***Participants***

Participants were 259 adults (*M*_age_ = 37.09, *SD*_age_ *=* 10.21; 52.51% women, 44.40% men, 3.09% non-specified gender) recruited for a 30-minute survey through Amazon’s Mechanical Turk and remunerated with $0.75. We restricted recruitment to participants who indicated they were currently employed in a workplace with at least three coworkers. We initially aimed to collect a sample of *N* = 250. A sensitivity analysis conducted in G*Power for a linear bivariate regression analysis (Faul et al., 2009)^^[[1]](#footnote-0)^^ revealed that a sample of *N =* 259 would allow us to detect a standardized regression coefficient of .17 or greater with 80% power and an alpha of .05.

***Procedure***

Participants completed a 26-item fit inventory (grouped and counterbalanced by subscale; see Table S2). After completing several exploratory measures of fluency, participants reported their current level of state authenticity and organizational commitment, as well as exploratory measures of approach and avoidance motivation (exploratory variables are not described here). Finally, participants self-reported demographics.

***Measures***

**Fit.** We measured fit with a series of 26 items (9 measuring self-concept fit, 8 measuring goal fit, 9 measuring social fit; see Table S2 for full item text). Participants rated their agreement with each item (1 = *strongly disagree*, 7 = *strongly agree*).

**State Authenticity.** We measured state authenticity with Lenton et al.’s (2013b) real-self overlap scale (RSOS). This scale uses a single-item pictorial measure of seven pairs of circles, with each pair varying in degree of overlap between them. The instructions read: “Please look at the pair of circles below. In each pair, the circle on the left represents who you feel yourself to be *IN YOUR WORKPLACE* and the circle on the right represents your *REAL SELF.* Your real self is who you *truly* are (which may not necessarily be the same as who you would like to be).” In each pair, one circle was labeled “me now,” and the other circle was labeled “real me.” Participants were instructed to select the circle pair that best represented how close they feel in their workplace to their true self.

**Organizational Commitment.** We measured organizational commitment with six items adapted from Cook and Wall (1980; e.g., “I would be very happy to spend the rest of my career with this organization;” “I often think about quitting,” reverse scored). Participants rated their agreement with each item (1 = *strongly disagree*, 6 = *strongly agree*). The composite showed excellent reliability, $\alpha$ = .90.

**Results**

***Evidence for Three Distinct Types of Fit***

**Factor Analysis*.*** We assessed the Kaiser-Meyer Olkin (KMO) Measure of Sampling Adequacy and conducted Bartlett’s Test of Sphericity to assess the data’s factorability. Both tests showed the data were factorable (KMO = .96, $\chi$^2^ (325) = 6154.85, *p* = .00). An initial principal components analysis yielded a three-components solution that explained 71.96% of the total variance. Similarly, the scree plot (see Figure S1) revealed the slope of the curve leveled off after three components.

**Figure S1.** *Scree Plot from a Principal Components Analysis of 26 Fit Items*


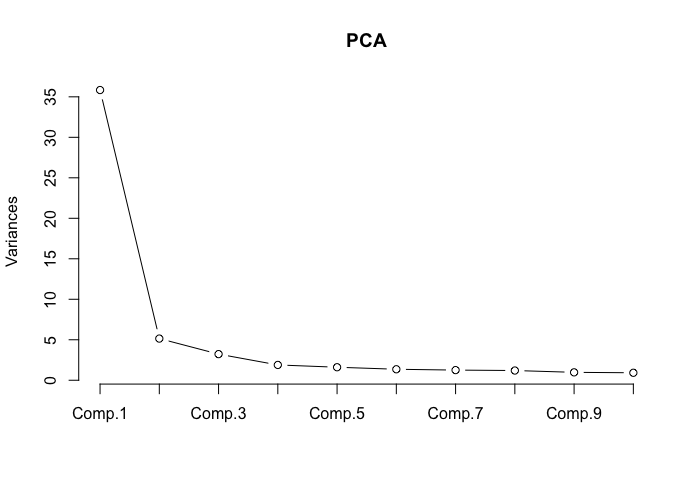


We also conducted a parallel analysis (Revelle, 2021), which suggested three factors (see Figure S2 and Table S1). We note that – while clearly above its simulated counterpart (eigenvalue = 0.50) – the third factor extracted through parallel analysis (eigenvalue = 0.95) fell just below the commonly applied Kaiser criterion (i.e., threshold of eigenvalue > 1). However, given the theoretical considerations of the SAFE model (and the very minor deviation from the cutoff as well as the superior performance over the simulated data), we opted to pursue a three-factor solution.

**Figure S2.** *Parallel Analysis of 26 Fit Items*


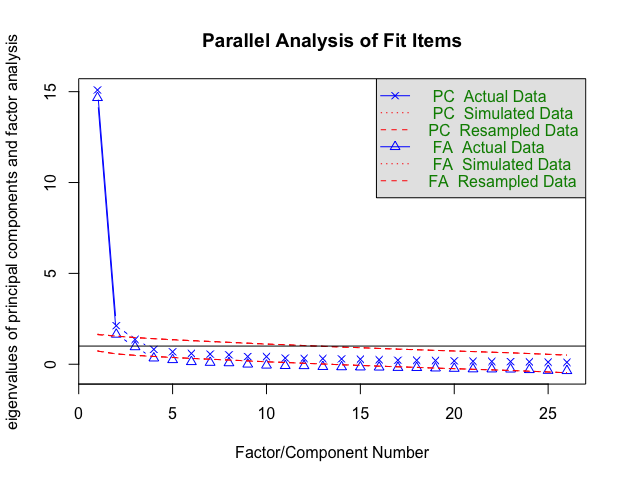


**Table S1**

*Results of Parallel Analysis Among Fit Items in the Pilot Study*

| Factor | Empirical Eigenvalue | Simulated Eigenvalue |
| --- | --- | --- |
| 1 | 14.67 | 0.75 |
| 2 | 1.64 | 0.58 |
| 3 | 0.95 | 0.50 |

We next subjected all 26 fit items to a principal axis factor analysis with oblique, promax rotation (Costello & Osborne, 2005). As seen in Table S2, the three types of fit showed excellent factor structure, with each type of fit loading onto a separate factor (all factor loadings > .40, with no cross-loadings > .30 on the 15-item scale; Table S2).

**Table S2**

*Results of Factor Analysis Among Fit Items in the Pilot Study*

| Item | Description | Factor 1 | | Factor 2 | Factor 3 |
| --- | --- | --- | --- | --- | --- |
| SCF 2 | **Even when I’m alone and doing nothing, simply being in my workplace makes me feel like myself.** | **.91** | -.08 | | .04 |
| SCF 3 | **Just being in my workplace suits the way I see myself.** | **.82** | .17 | | -.10 |
| SCF 8 | **My workplace feels true to who I am.** | **.80** | .05 | | .07 |
| SCF 1 | **Being in my workplace brings out my true self.** | **.79** | -.02 | | .09 |
| SCF 6 | **I feel ‘at home’ when I’m in my workplace.** | **.70** | .14 | | .06 |
| SCF 9 | When I am in my workplace, I generally feel in line with the ‘real me.’ | .70 | -.04 | | .27 |
| SCF 4 | My workplace is a good fit for who I am. | .64 | .33 | | -.08 |
| SCF 7 | I do NOT feel isolated from my inner self in my workplace. | .49 | .07 | | .19 |
| SCF 5 | I feel at ease with myself being in my workplace. | .41 | .30 | | .22 |
| GLF 3 | **My job is a place where I feel intrinsically motivated by my own goals.** | .01 | **.91** | | -.04 |
| GLF 5 | **Standards of success at my job match what I think it means to be successful.** | -.03 | **.88** | | -.04 |
| GLF 2 | **I often feel that my job is a place that allows me to realize my goals.** | .06 | **.82** | | -.03 |
| GLF 6 | **My behavior at work is motivated by things I value.** | -.02 | **.77** | | .01 |
| GLF 4 | **Tasks at my job are designed in a way that fits how I like to work.** | -.06 | .**75** | | .13 |
| GLF 7 | My job allows me to pursue topics and interests I am motivated to learn about. | .13 | .72 | | -.04 |
| GLF 8 | When working at my job, I feel like I am working toward my most prized goals. | .24 | .69 | | -.07 |
| GLF 1 | My workplace’s values are a good fit to my own personal values. | .10 | .67 | | .00 |
| SLF 9 | **When I’m around my coworkers, I feel like I am my true self.** | .14 | -.11 | | **.85** |
| SLF 6 | **I don’t feel like I need to be a different person around others at my job.** | .10 | -.22 | | **.85** |
| SLF 8 | **My coworkers do NOT judge me for being my true self.** | .04 | -.14 | | **.82** |
| SLF 7 | **I never have to hide the ‘real me’ when I’m with others at work.** | .07 | -.02 | | **.81** |
| SLF 5 | **I feel that people at my job understand exactly who I am.** | -.01 | .14 | | **.74** |
| SLF 4 | Other people at my job see me in the same way I see myself. | .06 | .07 | | .74 |
| SLF 1 | When I’m with other coworkers at my job, I feel I can by myself. | -.05 | .18 | | .71 |
| SLF 3 | I feel that other people at my job accept me for who I am. | -.18 | .39 | | .63 |
| SLF 2 | I feel connected to my coworkers. | -.04 | .27 | | .55 |

*Note.* SCF = Self-Concept Fit, GLF = Goal Fit, SLF = Social Fit. The five highest loading (or most face-valid, in cases where there was a tie) items on each factor are bolded.

**Descriptive Statistics and Internal Consistency*.*** Based on the results of this analysis, we selected the five highest-loading items from each factor, all with cross-loadings below .30 (Boateng et al., 2018; Costello & Osborne, 2005), and created a composite for each type of fit. For cases where there was a tie (i.e., between SLF 5 and SLF 4), we retained the item with higher face validity. Table S3 provides the overall means (SDs) and internal consistencies for all key variables measured in the pilot study. All three fit composites showed excellent reliability and did not correlate above *r =* .70^[[2]](#footnote-1)^.

***Tests of Predictive Validity***

The pilot study also provided an opportunity to carry out an exploratory test of the scale’s predictive validity by examining the unique relationships between each fit subscale and two key outcomes: state authenticity and organizational commitment.

**State Authenticity.** When we regressed state authenticity onto the three fit subscales simultaneously, the measures of self-concept fit (𝛽 = .50, *p* < .001) and social fit (𝛽 = .36, *p* < .001) both showed sizeable, unique relationships to state authenticity. Goal fit did not significantly predict state authenticity with the other two types of fit included in the model, and in fact showed a slight negative relationship (𝛽 = -.08, *p* = .23), suggesting a possible suppressor effect. The effect size for the relationship of self-concept fit and social fit, but not goal fit, to state authenticity was above the threshold of 𝛽 = .17 specified by sensitivity analyses. Together, the model explained over 50% of variance in state authenticity, *F*(3, 239) = 93.75, *p* < .001, *R*^2^_adj_ = .53.

**Table S3**

*Descriptive Statistics and Correlations Among Key Variables Measured in the Pilot Study*

|  | *M (SD)* | (1) | (2) | (3) | (4) |
| --- | --- | --- | --- | --- | --- |
| (1) Self-Concept Fit (α = .94) | 4.63 (1.45) |  |  |  |  |
| (2) Goal Fit (α = .91) | 5.06 (1.27) | .70*** |  |  |  |
| (3) Social Fit (α = .91) | 4.83 (1.39) | .70*** | .58*** |  |  |
| (4) State Authenticity (1 item) | 4.68 (1.45) | .69*** | .48*** | .66*** |  |
| (5) Org. Commitment (α = .90) | 3.86 (1.37) | .61*** | .68*** | .55*** | .55*** |

*Note.* **p* < .05. ***p* < .01. ****p* < .001.

**Organizational Commitment.** When we repeated this same simultaneous regression analysis with organizational commitment as the outcome, self-concept fit (𝛽 = .17, *p* = .018), goal fit (𝛽 = .48, *p* < .001), and social fit (𝛽 = .15, *p* = .024) each showed a unique predictive relationship to organizational commitment, although the direct effect of social fit fell below the threshold of 𝛽 = .17 specified by sensitivity analyses. Together, the three types of fit explained half of the variance in organizational commitment, *F*(3, 241) = 83.38, *p* < .001, *R*^2^_adj_ = .50.

**Discussion**

The results of this pilot study provided preliminary support for a new measure of fit that could be used to test several key propositions from the SAFE model by: (1) developing a highly reliable fit scale that empirically distinguishes among three fit constructs and (2) establishing the predictive validity of this measure by demonstrating that different types of fit uniquely predicts both state authenticity and interest in staying in versus leaving a setting (i.e., organizational commitment). Specifically, an exploratory factor analysis of a newly developed set of fit items supported a model positing three types of fit. Examining people’s feelings of fit at their place of work revealed that self-concept and social fit both uniquely predicted state authenticity and all three types of fit uniquely predicted one’s commitment to stay at one’s organization. Informed by the findings of this pilot study, we designed Study 1 to replicate and extend these preliminary findings in a different context: university students’ feelings of fit, authenticity, and commitment to their university. We adapted items used in this pilot study to refer to their university. In addition, we had some concerns that the mention of ‘true self’ in the item wording of some fit items and the authenticity measure could have artificially inflated their association. Thus, we made wording changes to four items in the fit scale to exclude mention of "true self" in an effort to reduce conceptual overlap with the authenticity measure. We also added a face valid measure of state authenticity in Study 1 to reduce this overlap.

**Study 1 Supplemental Materials**

Study 1 combined data across two waves of data collection from three separate samples that used largely overlapping measures and revealed very similar results. For the sake of parsimony and power, we report analyses on a combined dataset but provide a summary of analyses on the separate samples here.

**Method**

***Participants***

Sample A and B (collected during Wave 1 of data collection) included undergraduate students recruited from two large North American universities (*n =* 320 from University A in Canada, *n =* 219 from University B in the United States). Sample 3 (collected during Wave 2) was a preregistered (https://aspredicted.org/UVB_YUR) replication of Wave 1 with a broader sample of 430 U.S. university or college students (*M*_age_ = 23.19, *SD*_age_ = 6.22; 45.7% women, 44.1% men, 2.8% non-specified gender), recruited through Prolific Academic. A comparison of demographic characteristics across the three samples is provided in Table S4. Compared to Wave 1, Wave 2 had a higher proportion of male, White, Hispanic, and Black/African-American participants, but a lower proportion of Asian participants.

**Table S4**

*Demographics by Sample in Study 1*

| **Gender** | **Wave 1**  **University A**  *N* (%) | **Wave 1**  **University B**  *N* (%) | **Wave 1**  **Tota**l  *N* (%) | **Wave 2**  **Prolific Total**  *N* (%) |
| --- | --- | --- | --- | --- |
| Male | 103 (32.19%) | 51 (23.29%) | 154 (28.57%) | 207 (48.14%) |
| Female | 214 (66.88%) | 160 (73.06%) | 374 (69.39%) | 205 (47.67%) |
| Trans/non-binary | 3 (0.94%) | 6 (2.74%) | 9 (1.67%) | 14 (3.26%) |
| **Generation Status**  (First in family to attend college) | **Wave 1 University A**  *N* (%) | **Wave 1 University B**  *N* (%) | **Wave 1**  **Tota**l  *N* (%) | **Wave 2**  **Prolific Total**  *N* (%) |
| First Generation (Yes) | 40 (12.50%) | 34 (15.53%) | 74 (13.73%) | 87 (20.23%) |
| Non-First Generation (No) | 247 (77.19%) | 183 (83.56%) | 430 (79.78%) | 340 (79.07%) |
| **Racial/Ethnic Background** | **Wave 1 University A**  *N* (%) | **Wave 1 University B**  *N* (%) | **Wave 1**  **Tota**l  *N* (%) | **Wave 2**  **Prolific Total**  *N* (%) |
| East Asian | 104 (32.50%) | 66 (30.14%) | 170 (31.54%) | 40 (9.30%) |
| South Asian | 42 (13.13%) | 22 (10.05%) | 64 (11.87%) | 19 (4.42%) |
| Southeast Asian | 24 (7.50%) | 20 (9.13%) | 44 (8.16%) | 27 (6.28%) |
| Black/African/African  American | 9 (2.81%) | 4 (1.83%) | 13 (2.41%) | 44 (10.23%) |
| Hispanic/Latinx | 5 (1.56%) | 17 (7.76%) | 22 (4.08%) | 54 (12.56%) |
| Middle Eastern | 19 (5.94%) | 5 (2.28%) | 24 (4.45%) | 6 (1.40%) |
| Native American or Alaska  Native | 3 (0.94%) | 1 (0.46%) | 4 (0.74%) | 1 (0.23%) |
| Pacific Islander | 2 (0.63%) | 1 (0.46%) | 3 (0.56%) | 0 (0.00%) |
| White/European | 83 (25.94%) | 64 (29.22%) | 147 (27.27%) | 207 (48.14%) |
| Biracial/Mixed | 20 (6.25%) | 16 (7.31%) | 36 (6.68%) | 26 (6.05%) |
| Not Listed | 8 (2.50%) | 1 (0.46%) | 9 (1.67%) | 2 (0.47%) |
|  | **Wave 1 University A**  *M* (SD) | **Wave 1 University B**  *M* (SD) | **Wave 1**  **Total**  *M* (SD) | **Wave 2**  **Prolific Total**  *M* (SD) |
| **Age** (Years) | 21.01 (3.61) | 20.65 (2.39) | 20.86 (3.18) | 23.19 (6.22) |
| Between-Sample Comparison | *t*(534) = -1.29, *p* = .20, *d* = .11 | | *t*(960) = -7.51, *p* < .001, *d* = .49 | |
| **Family Social Class** (1-6 scale) | 4.24 (0.93) | 4.16 (1.16) | 4.21 (1.03) | 3.72 (1.12) |
| Between-Sample Comparison | *t*(534) = -0.90, *p* = .37, *d* = .08 | | *t*(959) = 7.04, *p* < .001, *d* = .46 | |

*Note.* Percentages do not sum to 100 due to missing data.

***Procedure and Measures***

Participants completed all measures listed below clustered by scale with scale order randomized. In addition to measures of self-concept, goal, and social fit, participants reported their state authenticity, university commitment, social belonging, autonomy, relatedness, competence, sense of self, and goal motivation, positive and negative affect, and socially desirable responding. Responses to demographics questions concluded the session. Because these samples were collected as part of broader projects with additional goals, additional variables were assessed that were not analyzed or reported for this project. Below is the full list of variables included in the two waves of data collection (with variations across wave noted in parentheses). Variables analyzed and reported in the manuscript are denoted with an asterisk.

**Conceptual Variables**

- Academic Difficulty
- Imposter Feelings
- Belonging*
- Academic Help-seeking (*one sub-scale not included in Wave 2*)
- Growth Mindset
- Self-efficacy (*only in Wave 2*)
- Competitiveness
- Collective Self-Esteem
- Self-Concept Fit*
- Goal Fit*
- Social Fit*
- State Authenticity (RSOS)* ^^[[3]](#footnote-2)^^
- State Authenticity (Single Item)*
- University Commitment*
- Autonomy, Competence, Relatedness *
- Positive/Negative Affect*
- Socially Desirable Responding*
- Sense of Self*
- Goal Motivation*

**Demographics**

- First-Generation Status
- Age
- Gender
- Semesters of College Completed
- Transfer Student Status
- Racial and/or Ethnic Background
- Mother Education
- Father Education
- Family Social Class
- Course Average
- University Name & Location (*only in Wave 2*)

**Table S5a**

*Descriptive Statistics and Correlations Among Variables Measured in Study 1 (Wave 1)*

|  | *M* (SD) | (1) | (2) | (3) | (4) | (5) | (6) | (7) | (8) | (9) | (10) | (11) | (12) | (13) | (14) | (15) |
| --- | --- | --- | --- | --- | --- | --- | --- | --- | --- | --- | --- | --- | --- | --- | --- | --- |
| (1) Self-Concept Fit (α = .94) | 4.44 (1.42) |  |  |  |  |  |  |  |  |  |  |  |  |  |  |  |
| (2) Goal Fit (α = .84) | 4.60 (1.18) | 0.64^***^ |  |  |  |  |  |  |  |  |  |  |  |  |  |  |
| (3) Social Fit (α = .89) | 4.55 (1.25) | 0.60^***^ | 0.45^***^ |  |  |  |  |  |  |  |  |  |  |  |  |  |
| (4) State Authenticity (Single-Item) | 4.89 (1.34) | 0.57^***^ | 0.50^***^ | 0.58^***^ |  |  |  |  |  |  |  |  |  |  |  |  |
| (5) University Commitment (α = .74) | 0.00 (0.81) | 0.59^***^ | 0.47^***^ | 0.38^***^ | 0.45^***^ |  |  |  |  |  |  |  |  |  |  |  |
| (6) Social Belonging (α = .85) | 0.01 (0.83) | 0.56^***^ | 0.51^***^ | 0.48^***^ | 0.48^***^ | 0.60^***^ |  |  |  |  |  |  |  |  |  |  |
| (7) Autonomy (α = .73) | 4.68 (0.85) | 0.40^***^ | 0.43^***^ | 0.50^***^ | 0.42^***^ | 0.35^***^ | 0.49^***^ |  |  |  |  |  |  |  |  |  |
| (8) Relatedness (α = .86) | 5.34 (0.94) | 0.36^***^ | 0.35^***^ | 0.52^***^ | 0.40^***^ | 0.35^***^ | 0.42^***^ | 0.62^***^ |  |  |  |  |  |  |  |  |
| (9) Competence (α = .73) | 4.45 (0.95) | 0.34^***^ | 0.39^***^ | 0.36^***^ | 0.38^***^ | 0.33^***^ | 0.48^***^ | 0.56^***^ | 0.52^***^ |  |  |  |  |  |  |  |
| (10) Positive Affect (α = .90) | 3.57 (0.65) | 0.63^***^ | 0.53^***^ | 0.47^***^ | 0.48^***^ | 0.50^***^ | 0.53^***^ | 0.49^***^ | 0.51^***^ | 0.48^***^ |  |  |  |  |  |  |
| (11) Negative Affect (α = .84) | 2.83 (0.71) | -0.33^***^ | -0.38^***^ | -0.33^***^ | -0.30^***^ | -0.32^***^ | -0.39^***^ | -0.43^***^ | -0.31^***^ | -0.38^***^ | -0.47^***^ |  |  |  |  |  |
| (12) BIDR-16 (SDE) (α = .69) | 3.78 (0.85) | 0.17^***^ | 0.25^***^ | 0.32^***^ | 0.28^***^ | 0.19^***^ | 0.34^***^ | 0.38^***^ | 0.21^***^ | 0.40^***^ | 0.30^***^ | -0.36^***^ |  |  |  |  |
| (13) BIDR-16 (IM) (α = .70) | 4.14 (0.94) | 0.15^***^ | 0.18^***^ | 0.24^***^ | 0.20^***^ | 0.14^**^ | 0.12^*^ | 0.21^***^ | 0.17^***^ | 0.15^***^ | 0.14^**^ | -0.18^***^ | 0.30^***^ |  |  |  |
| (14) Sense of Self (α = .85) | 3.82 (0.82) | 0.17^***^ | 0.24^***^ | 0.30^***^ | 0.29^***^ | 0.23^***^ | 0.43^***^ | 0.52^***^ | 0.42^***^ | 0.58^***^ | 0.31^***^ | -0.38^***^ | 0.62^***^ | 0.19^***^ |  |  |
| (15) Goal Motivation (α = .86) | 5.92 (0.96) | 0.23^***^ | 0.36^***^ | 0.27^***^ | 0.33^***^ | 0.22^***^ | 0.31^***^ | 0.38^***^ | 0.39^***^ | 0.48^***^ | 0.39^***^ | -0.18^***^ | 0.24^***^ | 0.19^***^ | 0.32^***^ |  |

*Note.* **p* < .05. ***p* < .01. ****p* < .001. BIDR = Balanced Inventory of Desirable Responding, IM = Impression Management, SDE = Self-Deceptive Enhancement. We standardized University Commitment and Social Belonging prior to forming composites due to items being on different scales.

**Table S5b**

*Descriptive Statistics and Correlations Among Variables Measured in Study 1 (Wave 2)*

|  | *M* (SD) | (1) | (2) | (3) | (4) | (5) | (6) | (7) | (8) | (9) | (10) | (11) | (12) | (13) | (14) | (15) |
| --- | --- | --- | --- | --- | --- | --- | --- | --- | --- | --- | --- | --- | --- | --- | --- | --- |
| (1) Self-Concept Fit (α = .94) | 4.58 (1.55) |  |  |  |  |  |  |  |  |  |  |  |  |  |  |  |
| (2) Goal Fit (α = .88) | 5.10 (1.19) | 0.70^***^ |  |  |  |  |  |  |  |  |  |  |  |  |  |  |
| (3) Social Fit (α = .89) | 4.44 (1.25) | 0.69^***^ | 0.60^***^ |  |  |  |  |  |  |  |  |  |  |  |  |  |
| (4) State Authenticity (Single-Item) | 4.97 (1.44) | 0.64^***^ | 0.55^***^ | 0.65^***^ |  |  |  |  |  |  |  |  |  |  |  |  |
| (5) University Commitment (α = .75) | 0.00 (0.81) | 0.65^***^ | 0.61^***^ | 0.49^***^ | 0.52^***^ |  |  |  |  |  |  |  |  |  |  |  |
| (6) Social Belonging (α = .87) | 0.00 (0.85) | 0.60^***^ | 0.60^***^ | 0.56^***^ | 0.58^***^ | 0.65^***^ |  |  |  |  |  |  |  |  |  |  |
| (7) Autonomy (α = .76) | 4.63 (0.97) | 0.41^***^ | 0.43^***^ | 0.48^***^ | 0.48^***^ | 0.37^***^ | 0.50^***^ |  |  |  |  |  |  |  |  |  |
| (8) Relatedness (α = .84) | 5.00 (1.03) | 0.48^***^ | 0.45^***^ | 0.48^***^ | 0.44^***^ | 0.45^***^ | 0.51^***^ | 0.59^***^ |  |  |  |  |  |  |  |  |
| (9) Competence (α = .77) | 4.54 (1.12) | 0.43^***^ | 0.48^***^ | 0.40^***^ | 0.48^***^ | 0.46^***^ | 0.60^***^ | 0.70^***^ | 0.58^***^ |  |  |  |  |  |  |  |
| (10) Positive Affect (α = .90) | 3.46 (0.72) | 0.63^***^ | 0.60^***^ | 0.53^***^ | 0.58^***^ | 0.60^***^ | 0.64^***^ | 0.49^***^ | 0.57^***^ | 0.59^***^ |  |  |  |  |  |  |
| (11) Negative Affect (α = .85) | 2.59 (0.78) | -0.37^***^ | -0.44^***^ | -0.37^***^ | -0.38^***^ | -0.44^***^ | -0.58^***^ | -0.45^***^ | -0.35^***^ | -0.50^***^ | -0.59^***^ |  |  |  |  |  |
| (12) BIDR-16 (SDE) (α = .75) | 3.84 (0.98) | 0.23^***^ | 0.27^***^ | 0.36^***^ | 0.39^***^ | 0.26^***^ | 0.47^***^ | 0.49^***^ | 0.36^***^ | 0.55^***^ | 0.38^***^ | -0.44^***^ |  |  |  |  |
| (13) BIDR-16 (IM) (α = .73) | 4.18 (1.01) | 0.14^***^ | 0.21^***^ | 0.29^***^ | 0.30^***^ | 0.20^**^ | 0.22^***^ | 0.20^***^ | 0.13^***^ | 0.26^***^ | 0.22^***^ | -0.27^***^ | 0.40^***^ |  |  |  |
| (14) Sense of Self (α = .88) | 3.79 (0.97) | 0.23^***^ | 0.28^***^ | 0.33^***^ | 0.42^***^ | 0.30^***^ | 0.50^***^ | 0.56^***^ | 0.43^***^ | 0.60^***^ | 0.37^***^ | -0.44^***^ | 0.67^***^ | 0.30^***^ |  |  |
| (15) Goal Motivation (α = .88) | 6.11 (0.92) | 0.31^***^ | 0.42^***^ | 0.35^***^ | 0.39^***^ | 0.37^***^ | 0.41^***^ | 0.49^***^ | 0.38^***^ | 0.52^***^ | 0.46^***^ | -0.38^***^ | 0.38^***^ | 0.20^***^ | 0.36^***^ |  |

*Note.* **p* < .05. ***p* < .01. ****p* < .001. BIDR = Balanced Inventory of Desirable Responding, IM = Impression Management, SDE = Self-Deceptive Enhancement. We standardized University Commitment and Social Belonging prior to forming composites, as responses scales were different.

**Study 1 Supplemental Results**

***Confirmatory Factor Analysis of Fit***

Table S5a and b summarizes descriptive statistics, scale reliabilities, and bivariate correlations for all variables in the two Waves of data collection. Given that an exploratory factor analysis in the pilot study yielded a three-factor solution, we used a confirmatory factor analysis (CFA) from the R package lavaan version 0.6-3 (Rosseel, 2012) to model each of the three types of fit as an interrelated latent construct, measured with the five respective fit items from each subscale. We used maximum likelihood estimation and full information maximum likelihood (FIML) to account for missing data. Here we report the results of this CFA on each Wave of data collection.

***CFA on Wave 1.*** The chi-square test of model fit was significant, $\chi$^2^ (87) = 276.73, *p* < .001, but this is to be expected for large samples (Bentler & Bonett, 1980; Curran et al., 2003). Other fit indices less biased by sample size suggested good model fit, CFI = .97, RMSEA = .06, SRMR = .04 (Clark & Watson, 2019; Finch & West, 1997; Hu & Bentler, 1999; Pituch & Stevens, 2015). Table S6 and Figure S3a summarizes the full CFA model with factor loadings and covariances between latent constructs. All subscale items loaded .62 or higher onto each latent factor.^[[4]](#footnote-3)^ This table also reveals that separate CFAs on Sample A and B (collected at different universities during Wave 1 of data collection), revealed very similar fit statistics and factor loadings.

The three fit factors were positively inter-correlated, with self-concept and goal fit showing the highest degree of overlap (*r =* .73). However, when comparing the hypothesized three-factor model to a simplified two-factor model that combined self-concept and goal fit items, the two-factor model showed poorer fit to the data ($\chi$^2^(76) = 613.55, *p* < .001, CFI = .89, RMSEA = .12, SRMR = .07) compared to the theoretically derived three-factor model ($\chi$^2^(87) = 276.73, *p* < .001, CFI = .97, RMSEA = .06, SRMR = .04).

**Table S6**

*Results of Confirmatory Factor Analysis for Full Sample in Study 1 (Wave 1) and by Site*

|  |  | | Standardized Factor Loadings | | | | |
| --- | --- | --- | --- | --- | --- | --- | --- |
| Latent Factor | Item | | Full Sample | | University A | | University B |
| Self-Concept Fit | SCF 1 | | .84 | | .84 | | .83 |
|  | SCF 2 | | .89 | | .90 | | .88 |
|  | SCF 3 | | .89 | | .89 | | .90 |
|  | SCF 4 | | .89 | | .90 | | .86 |
|  | SCF 5 | | .83 | | .83 | | .82 |
| Goal Fit | GLF 1 | | .82 | | .83 | | .81 |
|  | GLF 2 | | .62 | | .68 | | .51 |
|  | GLF 3 | | .84 | | .84 | | .84 |
|  | GLF 4 | | .70 | | .71 | | .67 |
|  | GLF 5 | | .63 | | .67 | | .59 |
| Social Fit | SLF 1 | | .85 | | .85 | | .85 |
|  | SLF 2 | | .84 | | .83 | | .87 |
|  | SLF 3 | | .68 | | .66 | | .69 |
|  | SLF 4 | | .82 | | .82 | | .84 |
|  | SLF 5 | | .74 | | .75 | | .74 |
|  |  | | Standardized Covariances | | | | |
| Latent Factors |  | | Full Sample | | University A | | University B |
| Self-Concept Fit, Goal Fit | | | .73 | | .72 | | .74 |
| Self-Concept Fit, Social Fit | | | .64 | | .61 | | .71 |
| Goal Fit, Social Fit | | | .50 | | .47 | | .57 |
|  |  | Model Fit Statistics | | | | | |
|  |  | Full Sample | | University A | | University B | |
| $\chi$^2^ (87) |  | 276.73, *p* < .001 | | 207.27, *p* < .001 | | 178.08, *p* < .001 | |
| CFI |  | .97 | | .96 | | .96 | |
| RMSEA |  | .06 | | .07 | | .07 | |
| SRMR |  | .04 | | .05 | | .04 | |

*Note.* SCF = Self-Concept Fit, GLF = Goal Fit, SLF = Social Fit.

**
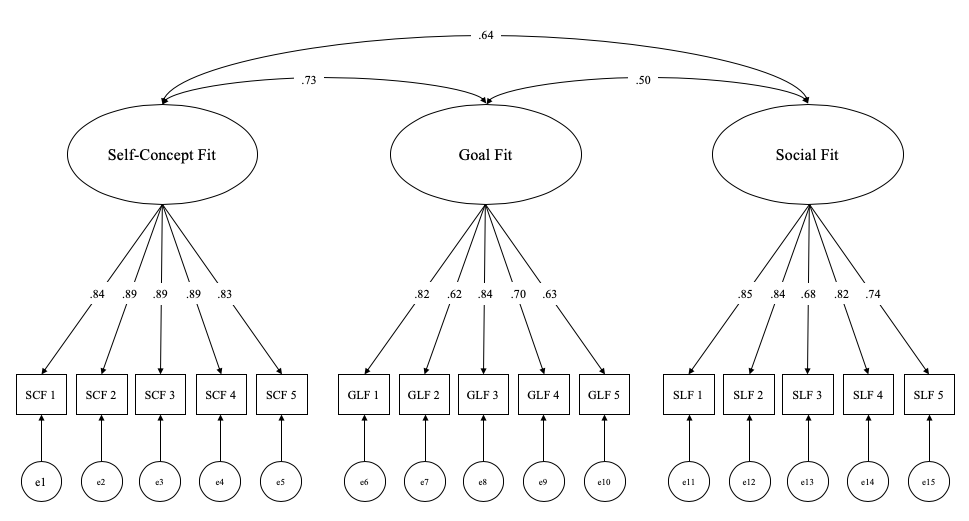
**

**Figure S3a**. *Confirmatory Factor Analysis of Fit Measures in Study 1 (Wave 1).*

**
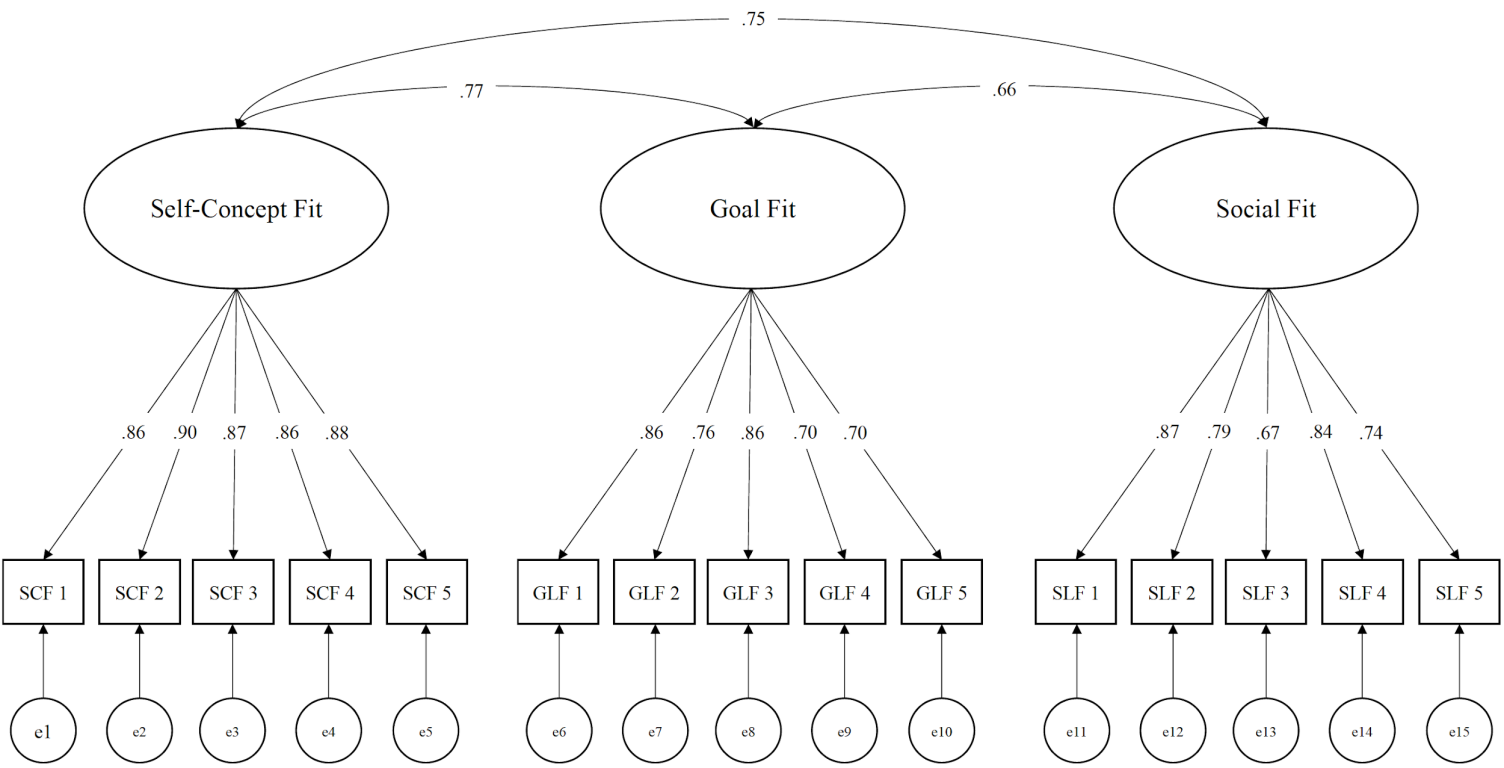
**

**Figure S3b**. *Confirmatory Factor Analysis of Fit Measures in Study 1 (Wave 2).*

***CFA on Wave 2.*** In Wave 2, again the chi-square test of model fit was significant, $\chi$^2^ (87) = 183.21, *p* < .001, but other fit indices less biased by sample size indicated good model fit; CFI = .98, RMSEA = .05, SRMR = .03 (Clark & Watson, 2019; Finch & West, 1997; Hu & Bentler, 1999; Pituch & Stevens, 2015). All subscale items loaded .67 or higher onto each latent factor (See Figure S3b). The correlations among the three fit factors were slightly larger than in Wave 1, with self-concept and goal fit again showing the highest overlap (*r =* .77). As in Wave 1, however, a two-factor model (combining self-concept and goal fit items) had poorer fit to the data ($\chi$^2^ (89) = 553.47, *p* < .001, CFI = .90, RMSEA = .11, SRMR = .06) compared to the three-factor model ($\chi$^2^ (87) = 183.21, *p* < .001, CFI = .98, RMSEA = .05, SRMR = .03).

***Distinguishing Fit from State Authenticity and Belonging.***  In the full sample (combining Wave 1 and 2), we also conducted an exploratory factor analysis (EFA) to examine whether the three types of fit are also distinct from both state authenticity and belongingness (5 for self-concept fit, 5 for goal fit, 5 for social fit, 2 for state authenticity, and 4 for belongingness). The self-determination (SDT) items were not included in these analyses because those items failed to factor analyze into their theoretically defined three-factor structure in an initial analysis (contact authors for details).

First, both a Kaiser-Meyer Olkin (KMO) Measure of Sampling Adequacy and Bartlett’s Test of Sphericity showed that the data were factorable (KMO = .96, *χ*^2^ (210) = 14428.22, *p* < .001). Moreover, an exploratory factor analysis (parallel analysis, Revelle, 2021) revealed the expected five factor solution (Figure S4). We then subject all items to a principle axis factor analysis with oblique, oblimin rotation. With a cut-off at 0.40, three types of fit, authenticity, and belongingness loaded separately onto different factors (see Table S7). These analyses confirm that our three measures of fit are empirically as well as conceptually distinct from our measure of state authenticity and from a measure of belonging.


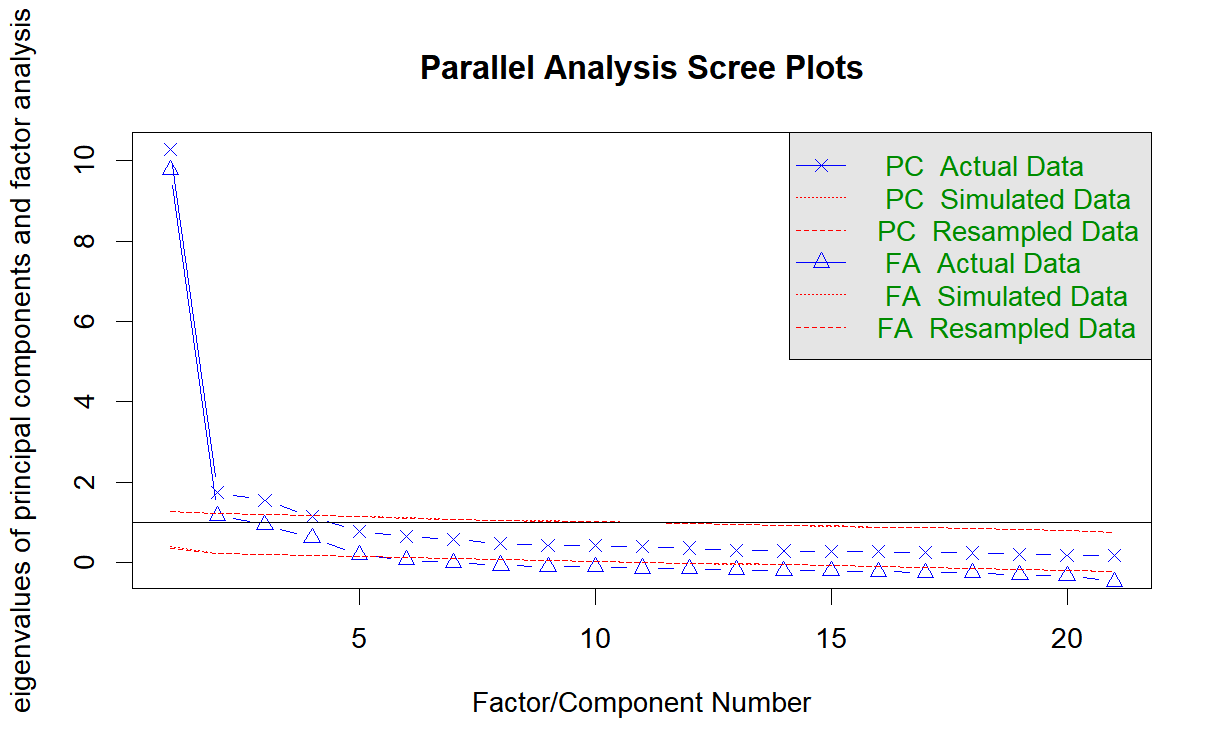


**Figure S4.** *Parallel Analysis of Fit, Authenticity, and Belongingness Items*

**Table S7**

*Results of Factor Analysis Among Fit, Authenticity, and Belongingness Items*

| Item | Factor 1 | Factor 2 | Factor 3 | Factor 4 | Factor 5 |
| --- | --- | --- | --- | --- | --- |
| SCF1 | **.91** | .02 | -.04 | -.05 | -.04 |
| SCF2 | **.85** | .02 | .06 | .02 | -.05 |
| SCF3 | **.78** | .00 | .11 | .06 | -.01 |
| SCF4 | **.73** | .08 | .07 | -.04 | .10 |
| SCF5 | **.81** | .03 | -.04 | .04 | .09 |
| GLF1 | -.04 | .00 | **.83** | .00 | .09 |
| GLF2 | .02 | .03 | **.71** | .01 | -.11 |
| GLF3 | .07 | -.01 | **.80** | -.01 | .03 |
| GLF4 | .01 | .01 | **.60** | .06 | .14 |
| GLF5 | .02 | .05 | **.68** | .04 | -.15 |
| SLF1 | .10 | **.75** | -.01 | .05 | .04 |
| SLF2 | -.06 | **.85** | -.05 | .05 | .07 |
| SLF3 | .02 | **.69** | .06 | .03 | -.16 |
| SLF4 | .00 | **.82** | .05 | -.04 | .02 |
| SLF5 | .21 | **.56** | .08 | -.01 | .01 |
| Belong1 | -.05 | -.04 | .08 | **.81** | -.02 |
| Belong2 | -.05 | .11 | -.04 | **.83** | -.02 |
| Belong3 | .24 | .01 | .03 | **.59** | .08 |
| Belong4 | .39 | -.04 | .07 | **.48** | .14 |
| Auth1 | .11 | .25 | .21 | .06 | **.42** |
| Auth2 | .08 | .23 | .07 | .08 | **.46** |

Note. SCF = Self-Concept Fit, GLF = Goal Fit, SLF = Social Fit, Belong = Belongingness, Auth = State Authenticity. Those loadings above the .40 cut-off are bolded.

***Which Types of Fit Predict Students’ State Authenticity and University Commitment?***

***Predicting State Authenticity from Fit.*** Core analyses in the main paper present results collapsed across wave/sample. Here we describe them separated. Consistent with the SAFE model, all three types of fit significantly and uniquely predicted state authenticity both in Wave 1: self-concept fit, 𝛽 = .23, *p* < .001; goal fit, 𝛽 = .19, *p* < .001; and social fit, 𝛽 = .35, *p* < .001 (explaining 42% of the variance in state authenticity, *F*(3, 491) = 121.70, *p* < .001, *R*^2^_adj_ = .42); and in Wave 2: self-concept fit, 𝛽 = .33, *p* < .001; goal fit, 𝛽 = .13, *p* < .001; social fit, 𝛽 = .34, *p* < .001 (explaining 50% of the variance in state authenticity, *F*(3, 425) = 144.8, *p* < .001, *R*^2^_adj_ = .50.

***Predicting University Commitment from State Authenticity.*** In addition, we regressed university commitment simultaneously on all three fit measures both in Wave 1 (with Sample A/B as a covariate, which did not moderate effects) and Wave 2. In Wave 1, both self-concept fit, 𝛽 = .46, *p* < .001, and goal fit, 𝛽 = .14, *p* = .004 (but not social fit, 𝛽 = .03, *p* = .548) significantly and uniquely predicted university commitment (explaining 36% of the variance in university commitment, *F*(4, 481) = 70.22, *p* < .001, *R*^2^_adj_ = .36). Similarly, in Wave 2, self-concept fit, 𝛽 = .43, *p* < .001; and goal fit, 𝛽 = .30, *p* < .001 (but not social fit, 𝛽 = .005, *p* = .928) significantly predicted university commitment (explaining 47% of the variance in university commitment, *F*(4, 425) = 125, *p* < .001, *R*^2^_adj_ = .47).

***Does Fit Predict University Commitment via State Authenticity?*** Finally, path analyses testing the unique relationship between each type of fit and university commitment as statistically mediated through state authenticity using the R package lavaan version 0.6-3 (Rosseel, 2012) also revealed similar effects in each Wave (see Figure S5a and b). For Wave 1 analyses, Sample A/B was included as a significant covariate only in paths predicting commitment (but not from each type of fit to authenticity).

In Wave 1 analyses, there was a significant indirect effect of self-concept fit (*a***b =* .02, *p* = .008), goal fit (*a*b =* .02, *p =* .01), and social fit (*a*b =* .03, *p* = .003). Self-concept fit (𝛽 = .25, *p* < .001), but neither goal fit (𝛽 = .06, *p* = .067) nor social fit (𝛽 = -.01, *p* = .786), retained direct effects to university commitment with state authenticity in the model. In Wave 2 analyses, we replicated the significant indirect effect of self-concept fit (*a***b =* .02, *p* = .016) and social fit (*a*b =* .03, *p* = .014); but the indirect effect of goal fit was marginal (*a*b =* .01, *p =* .059). With state authenticity in the model, self-concept fit (𝛽 = .20, *p* < .001) and goal fit (𝛽 = .19, *p* < .001), but not social fit (𝛽 = -.02, *p* = .45), retained direct effects to university commitment.


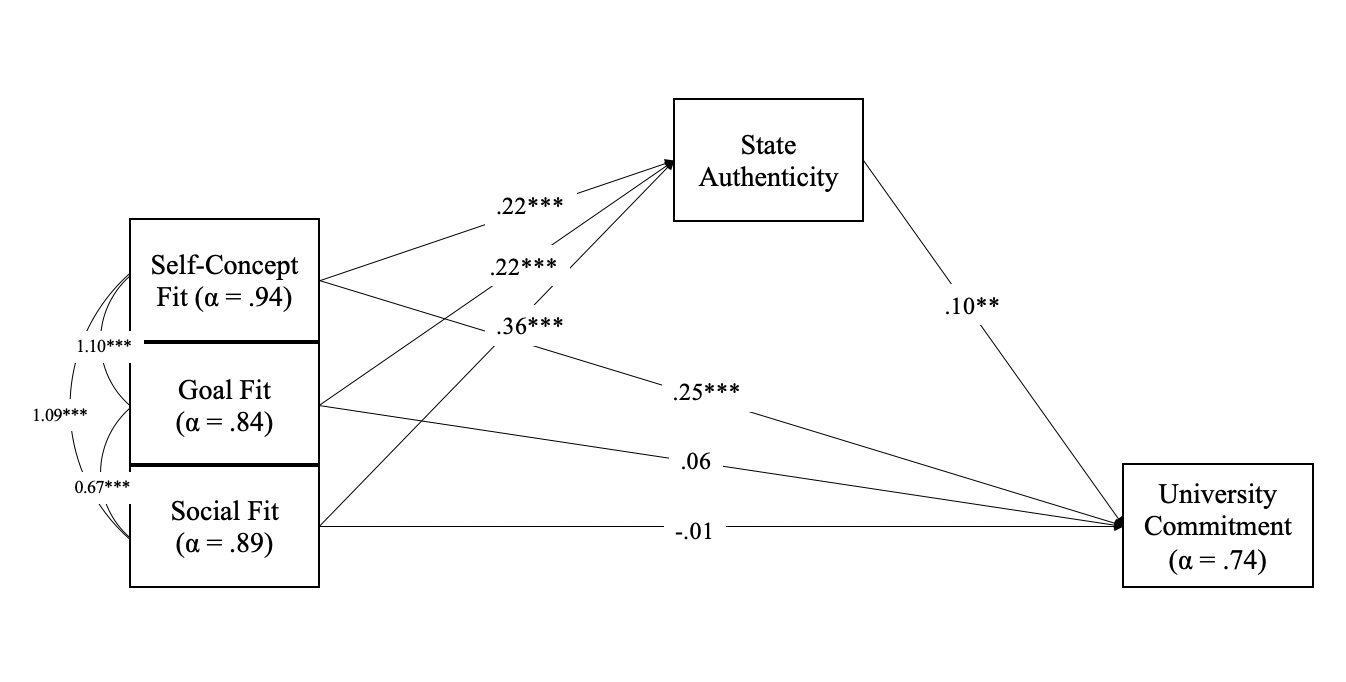


**Figure S5a**. Study 1 (Wave 1): *Relation of Each Type of Fit to Organizational Commitment as Mediated Via State Authenticity. Path Coefficients Reflect Standardized Betas; Relationships Among Fit Constructs Reflect Raw Covariances (i.e., Their Estimates May Surpass 1.00)*


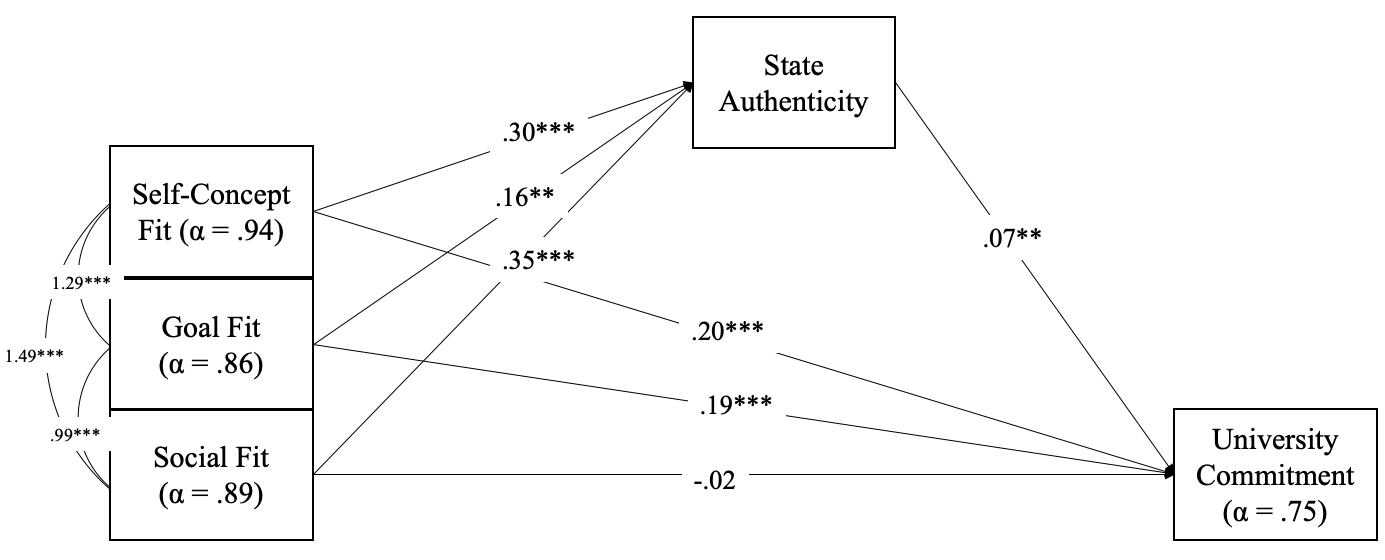


**Figure S5b**. Study 1 (Wave 2): *Relation of Each Type of Fit to University Commitment as Mediated by State Authenticity. Path Coefficients Reflect Standardized Betas; Relationships Among Fit Constructs Reflect Raw Covariances (i.e., Their Estimates May Surpass 1.00)*

***Tests of Convergent and Discriminant Validity***

The main manuscript provides tests of convergent and discriminant validity on the combined sample as well as analyses ruling out alternative explanations for the links between fit and authenticity. Here we provide these analyses separately by each Wave of data analysis. As summarized in Table S8, the results of these analyses yielded similar results for each Wave of data collection (note also that there was no evidence that results from Wave 1 were moderated by Sample A/B). The one exception was that in Wave 2, the relationship between goal fit and state authenticity (which had a weaker overall effect size in Wave 2) was not robust to controlling for other related constructs.

**Table S8**

*Results of Models Testing Discriminant Validity by Controlling for Conceptually Related Variables When Regressing State Authenticity onto Fit Measures in Study 1 (Wave 1 and 2)*

|  | **Original Model**  **(Model 0)** | | | **Self-Determination**  **Theory (Model 1)** | | | **Motivation, Self, & Belonging (Model 2)** | | **Positive & Negative**  **Affect (Model 3)** | | **Social Desirability**  **(Model 4)** | |
| --- | --- | --- | --- | --- | --- | --- | --- | --- | --- | --- | --- | --- |
|  | **Wave 1** | **Wave 2** | **Wave 1** | | **Wave 2** | **Wave 1** | | **Wave 2** | **Wave 1** | **Wave 2** | **Wave 1** | **Wave 2** |
| Self-Concept Fit | .23*** | .33*** | .23*** | | .30*** | .23*** | | .31*** | .18*** | .25*** | .26*** | .36*** |
| Goal Fit | .19*** | .13** | .15** | | .07 | .10* | | .03 | .16*** | .06 | .15*** | .10* |
| Social Fit | .35*** | .31*** | .30*** | | .28*** | .28*** | | .25*** | .33*** | .31*** | .32*** | .25*** |
| Competence | - | - | .09* | | .13* | - | | - | - | - | - | - |
| Autonomy | - | - | .05 | | .10* | - | | - | - | - | - | - |
| Relatedness | - | - | .03 | | .02 | - | | - | - | - | - | - |
| Sense of Self | - | - | - | | - | .08 | | .14*** | - | - | - | - |
| Goal Motivation | - | - | - | | - | .10* | | .13*** | - | - | - | - |
| Belonging | - | - | - | | - | .10* | | .13** | - | - | - | - |
| Positive Affect | - | - | - | | - | - | | - | .12* | .20*** | - | - |
| Negative Affect | - | - | - | | - | - | | - | -.14 | -.06 | - | - |
| BIDR (SDE) | - | - | - | | - | - | | - | - | - | .08* | .17*** |
| BIDR (IM) | - | - | - | | - | - | | - | - | - | .04 | .10** |

Note. **p* < .05. ***p* < .01. ****p* < .001. BIDR = Balanced Inventory of Desirable Responding, IM = Impression Management, SDE = Self-Deceptive Enhancement.

***Study 1: Comparisons by Race/Ethnicity***

Exploratory analyses of the combined sample compared fit and authenticity by race/ethnicity (Table S9). Compared with their White peers, non-White participants reported significantly lower levels of self-concept fit, goal fit, and state authenticity (but equivalent levels of social fit).

**Table S9**

*Descriptive Statistics for Fit and Authenticity by Race in Study 1*

|  |  | Descriptive Statistics | | |
| --- | --- | --- | --- | --- |
| Latent Factor | Non-White (*n*= 608)  *M* (*SD*) | White (*n*=354)  *M* (*SD*) | Cohen’s *d* | *t*-test |
| Self-Concept Fit  Goal Fit  Social Fit | 4.46 (1.46) | 4.74 (1.47) | .19 | -2.84** |
|  | 4.79 (1.20) | 5.01 (1.16) | .18 | -2.76** |
|  | 4.49 (1.30) | 4.47 (1.35) | .01 | 0.15 |
| State Authenticity  RSOS  Combined Measure | 4.66 (1.45) | 4.79 (1.43) | .09 | -1.39 |
|  | 4.85 (1.40) | 5.05 (1.36) | .15 | -2.21* |
|  | 4.75 (1.26) | 4.93 (1.24) | .14 | -2.12* |
| *Note.* **p* < .05. ***p* < .01. ****p* < .001. | | | | |

**Study 2: Supplemental Measures**

***Additional Measures for Study 2***

Although the focus of Study 2 was the within-person effects described in the main manuscript, this study included additional measures intended for other sets of pre-registered hypotheses. The following measures were included in the T1 baseline survey (and, in some cases, again in the T2 survey): Acculturation Level (Vancouver Index of Acculturation; Ryder, Alden, Paulhus, 2000), Personality (Big Five Inventory – 2 Short Form; John & Soto, 2015), Academic Motivation (Academic Motivation Scale-College; Vallerand, et al., 1992), University Commitment, and Cross-Cultural Friendship Similarity.

***Study 2: Sample Stimuli for Working Memory Capacity Measure***

Participants received the following instructions in the T1 baseline survey to familiarize them with the working memory capacity measure.

| 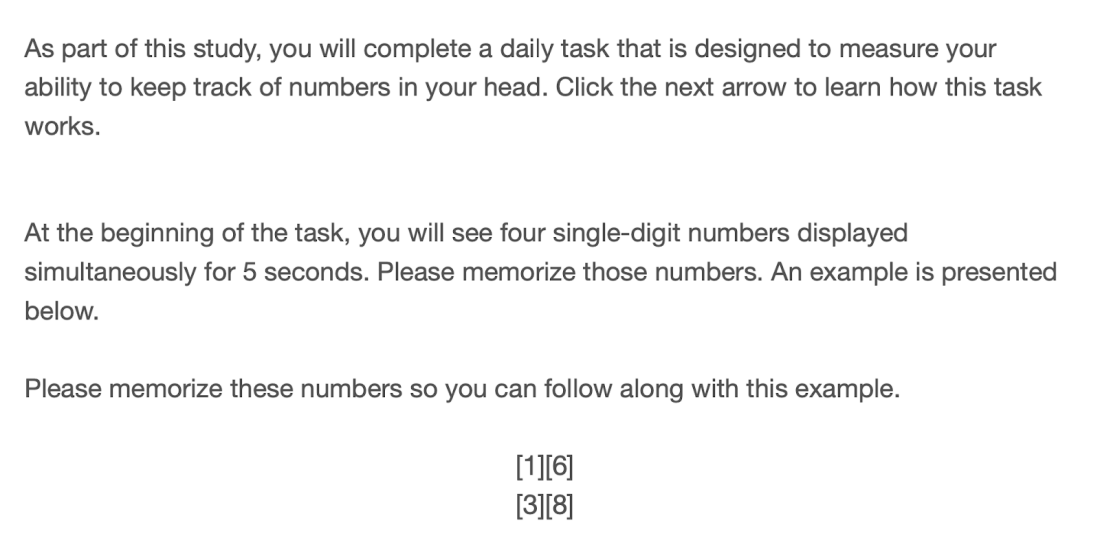  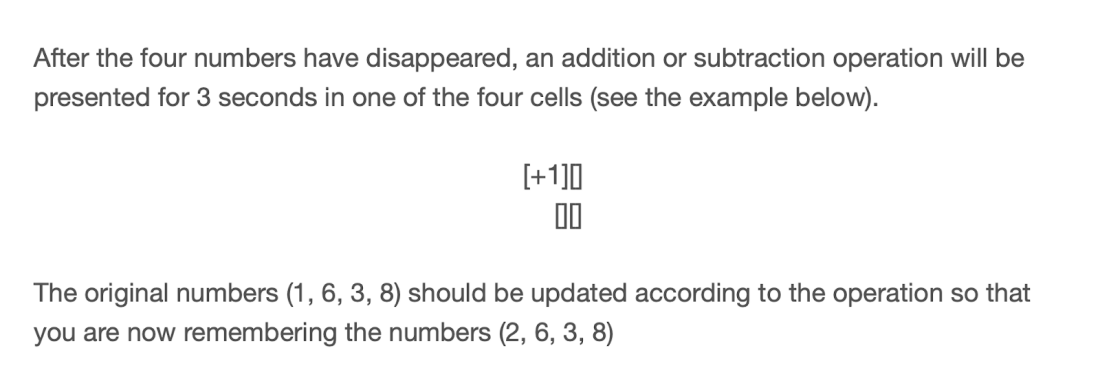  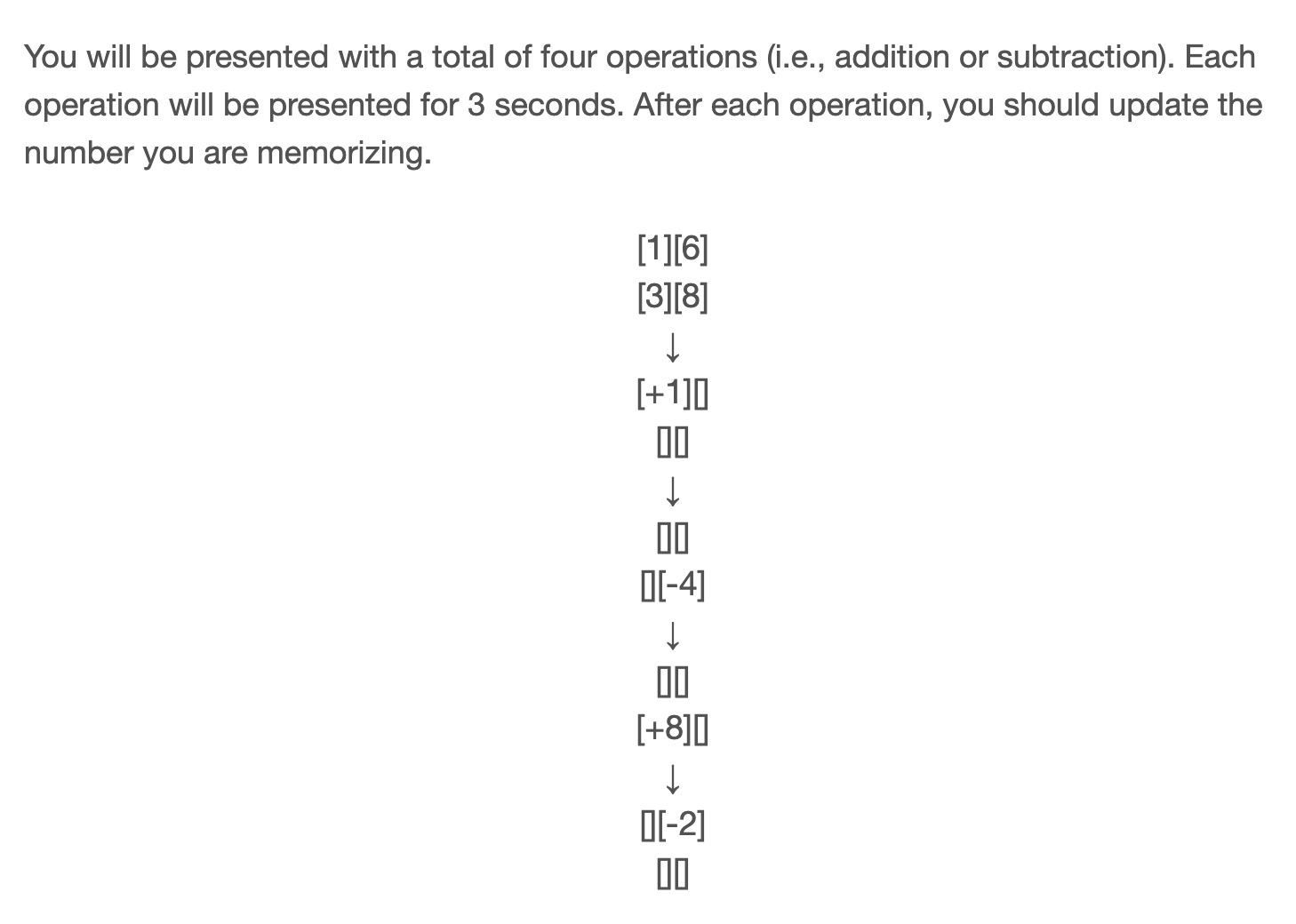  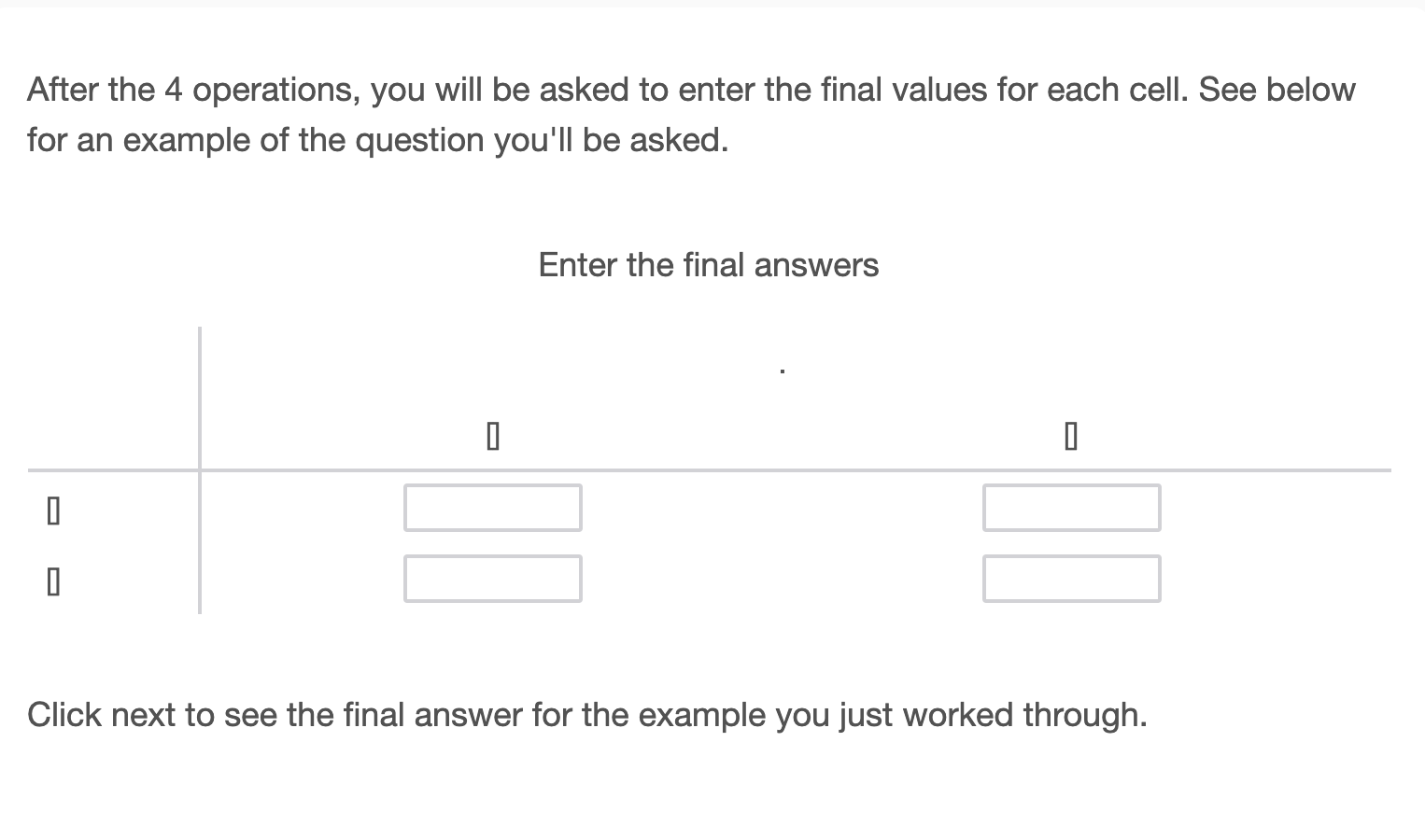  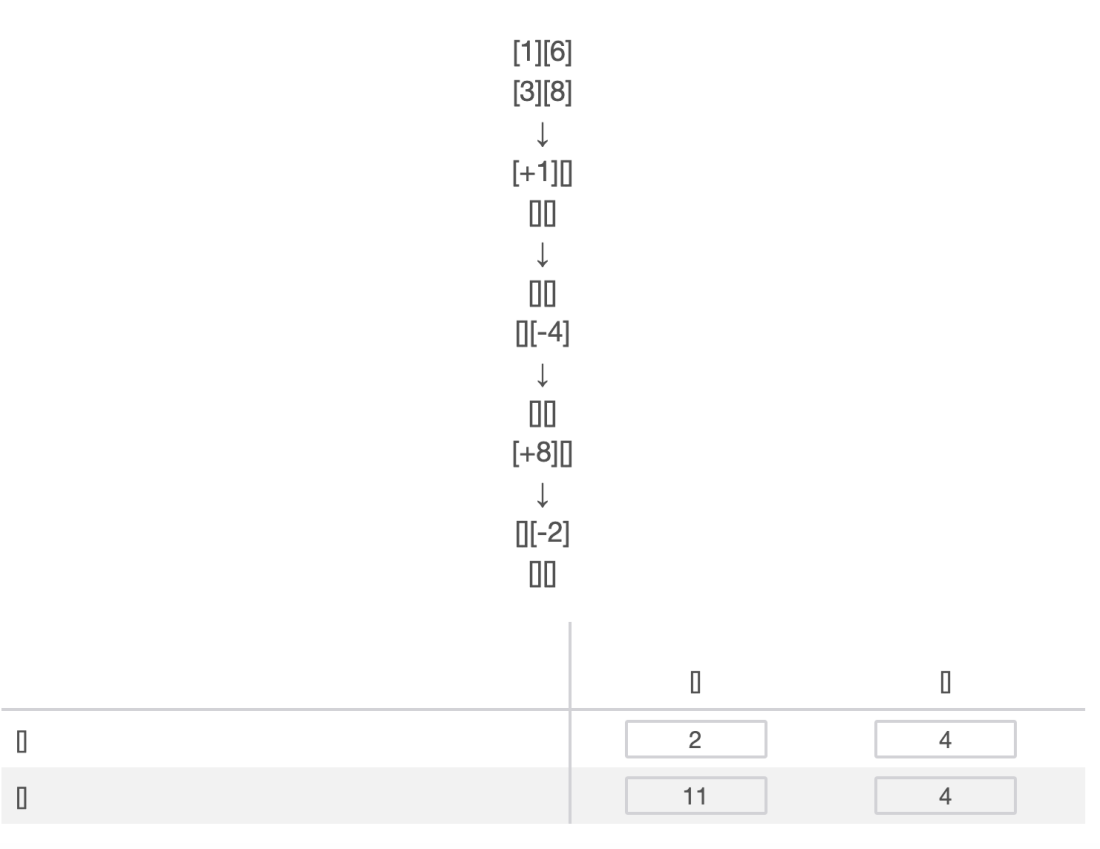 |
| --- |

**Study 2: Supplemental Results**

The primary focus of Study 2 was the within-person effects that reflect the state-specificity of fit and authenticity predicting outcomes. Table S10 provides a summary of the between-person effects from these same analyses. Both within- and between-person variability in authenticity predicted each of the outcomes (except working memory capacity). For measures of fit, within-person variability in fit predicted outcomes more consistently than between-person variability.

Exploratory analyses similar as in Study 1 compared fit and authenticity by race/ethnicity (Table S11). Contrast to Study 1, we did not find significant difference in fit or state authenticity between White vs. Non-White participants, although the means are in the predicted direction.

**Table S10**

*Study 2 Within-Person & Between-Person Results of Momentary Fit and Authenticity Predicting Momentary Outcomes*

|  | **Willingness to Return** | | **State Attachment**  **to University** | | **Working Memory Capacity** | | **Emotional Burnout** | |
| --- | --- | --- | --- | --- | --- | --- | --- | --- |
|  | Within-Person | Between-Person | Within-Person | Between-Person | Within-Person | Between-Person | Within-Person | Between-Person |
| **Predicting Outcomes from Fit** | | | | | | | | |
| Momentary Self-Concept Fit | β = .16*** | β = .45*** | β = .10*** | β = .11 | β = .12** | β = .19 | β = -.12*** | β = -.10 |
| Momentary Goal Fit | β = .11*** | β = .04 | β = .13*** | β = .34*** | β = -.04 | β = -.08 | β = -.03 | β = -.06 |
| Momentary Social Fit | β = .15*** | β = .12 | β = .07** | β = .31*** | β = -.08* | β = -.10 | β = -.04 | β = -.11 |
| **Predicting Outcomes from Authenticity** | | | | | | | | |
| State Authenticity | β = .23*** | β = .38*** | β = .36*** | β = .79*** | β = -.01 | β = -.05 | β = -.23*** | β = -.14* |

Table S11

*Study 2 Exploratory Analyses Comparing Fit and State Authenticity by Race/Ethnicity*

|  | **White**  **(*n* = 47)**  ***M* (SD)** | **Non-White**  **(*n* = 206)**  ***M* (SD)** | ***t*** | ***p*** |
| --- | --- | --- | --- | --- |
| Self-concept fit | 5.29 (0.90) | 5.07 (0.89) | t(68.01)=-1.50 | 0.14 |
| Goal fit | 5.24 (0.91) | 5.12 (0.91) | t(68.73)=-0.79 | 0.43 |
| Social fit | 5.17 (1.15) | 4.93 (1.10) | t(66.51)=-1.29 | 0.20 |
| State Authenticity | 5.13 (1.12) | 4.95 (1.07) | t(66.49)=-1.01 | 0.32 |

1. A sensitivity analysis conducted in G*Power for a multiple regression model with three predictors (i.e., types of fit) yielded the same effect size threshold, *r =* .17. [↑](#footnote-ref-0)
2. Further CFA analysis showed that a simplified two-factor model combining self-concept and goal fit items has poorer fit to the data (χ^2^[103] = 641.32, p < .001, CFI = .85, RMSEA = .15, SRMR = .07) than the theoretically-derived three-factor model (χ^2^[101] = 328.94, p < .001, CFI = .93, RMSEA = .10, SRMR = .05). We thus retain the theoretical three-factor model. [↑](#footnote-ref-1)
3. In addition to the face-valid authenticity item reported in the main manuscript, we also included a single-item pictorial measure (RSOS; Lenton, Bruder et al., 2013), adapted to refer to participants’ university. This visual measure of authenticity was positively correlated with the face-valid item, *r =* .58, *p* < .001, and effects using a combined measure were the same. However, we report results using only the face valid measure because of concerns of construct overlap with fit measures mentioning ‘real self’. [↑](#footnote-ref-2)
4. Examining residual correlations suggested that the item SLF 5 (“I feel that people at [University] understand exactly who I am”) showed modest overlap with items from the self-concept fit subscale. Because excluding this item revealed no significant drop in model fit, $\Delta\chi$^2^ (4) = -44.39, *p* = 1.00; we retained it in the social fit subscale, acknowledging its shared variance with the self-concept fit subscale. Additionally, conducting the CFA separately by each university showed largely similar fit indices and factor loadings (results provided in this SOM), suggesting no substantial variation by site. [↑](#footnote-ref-3)
